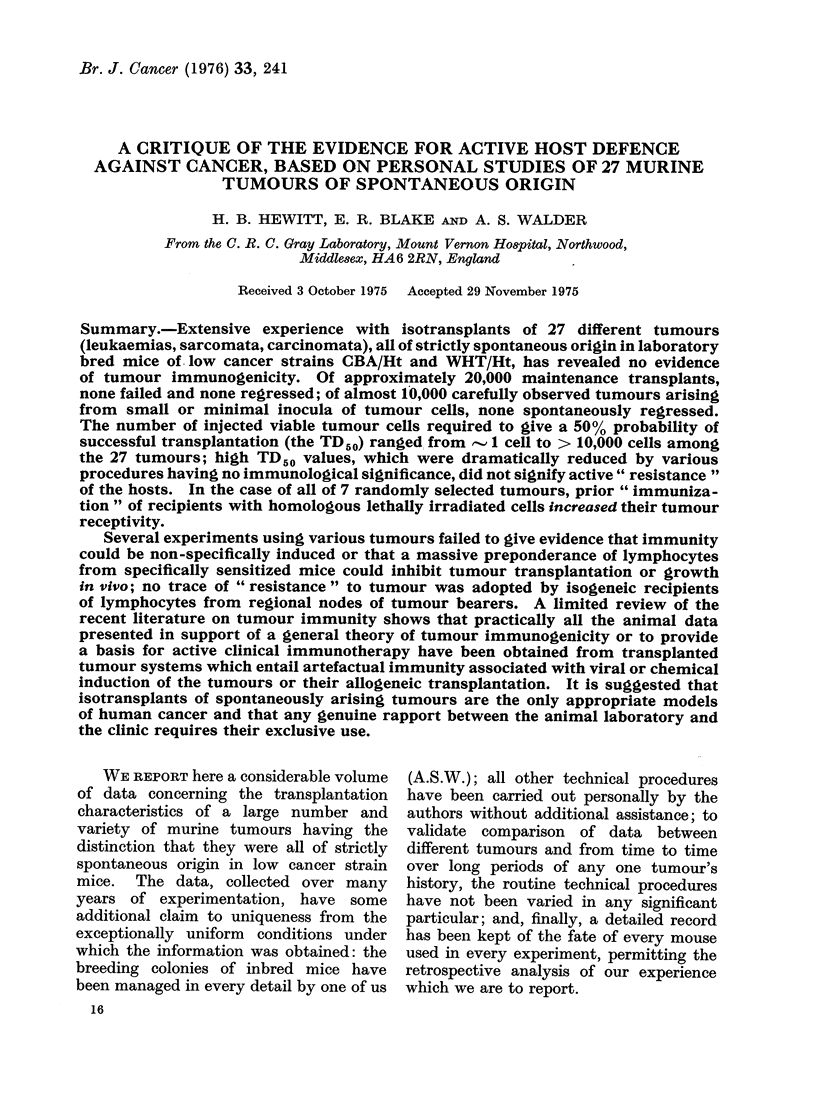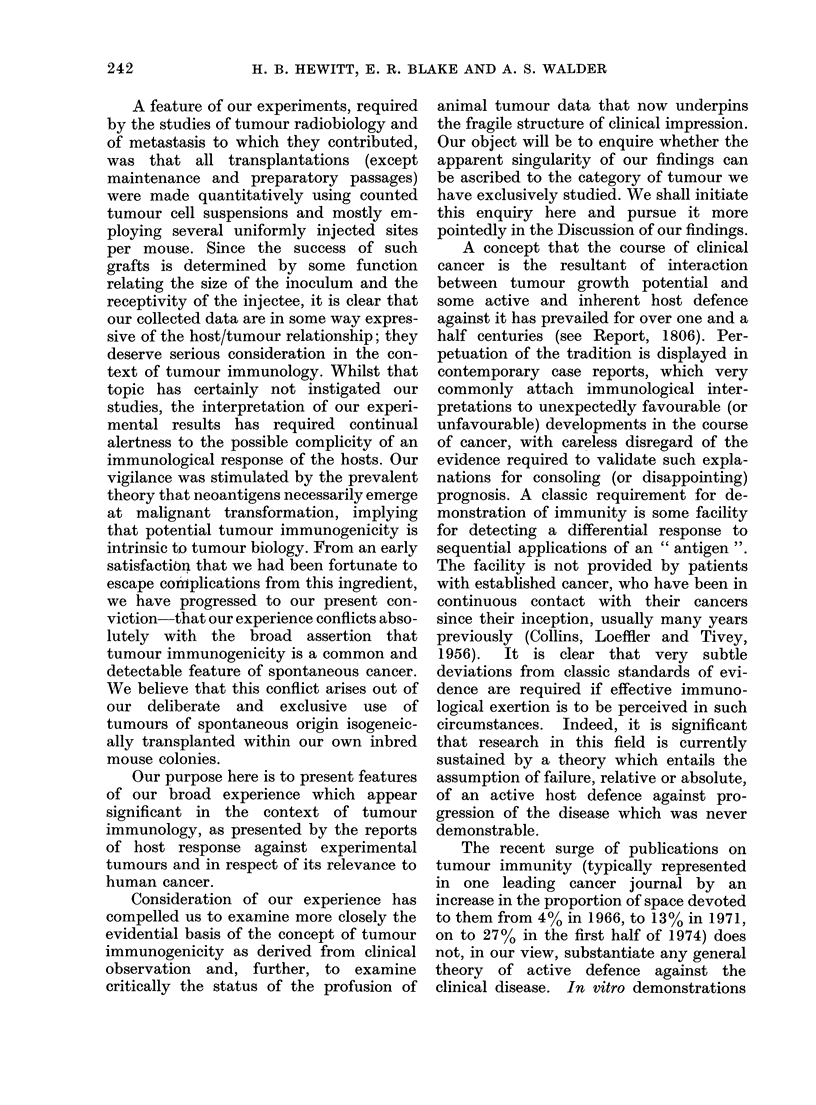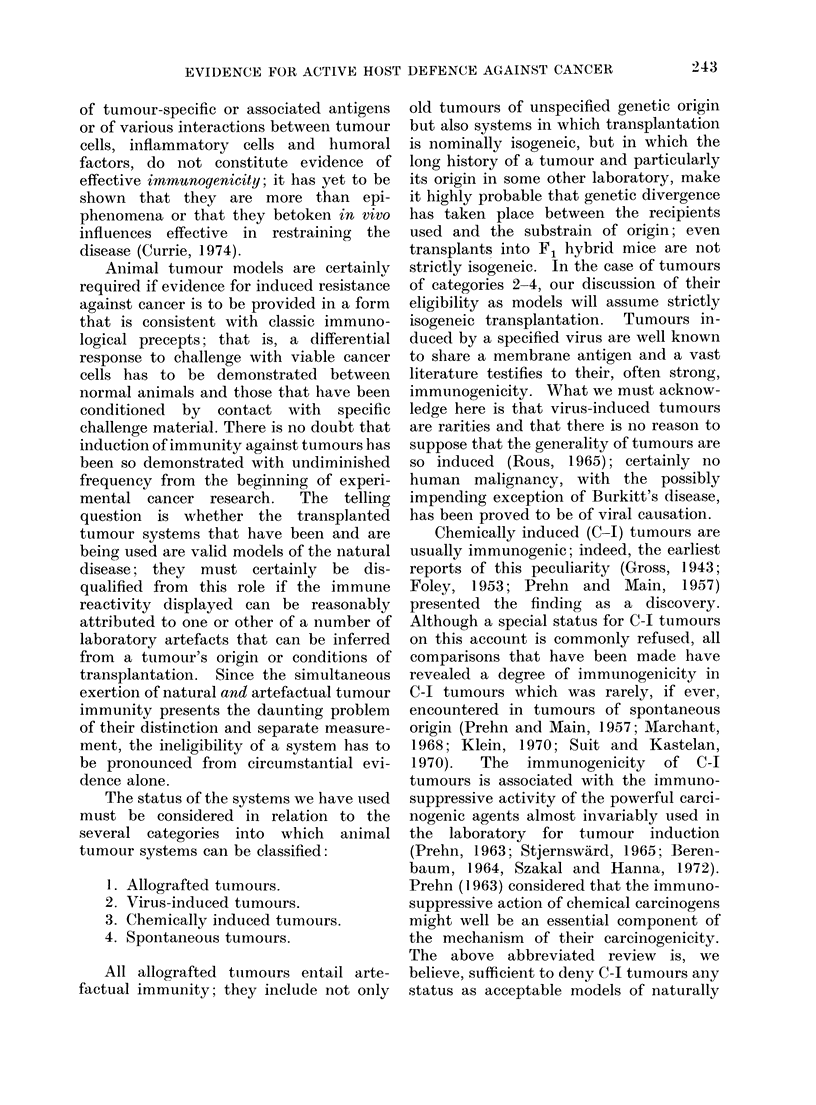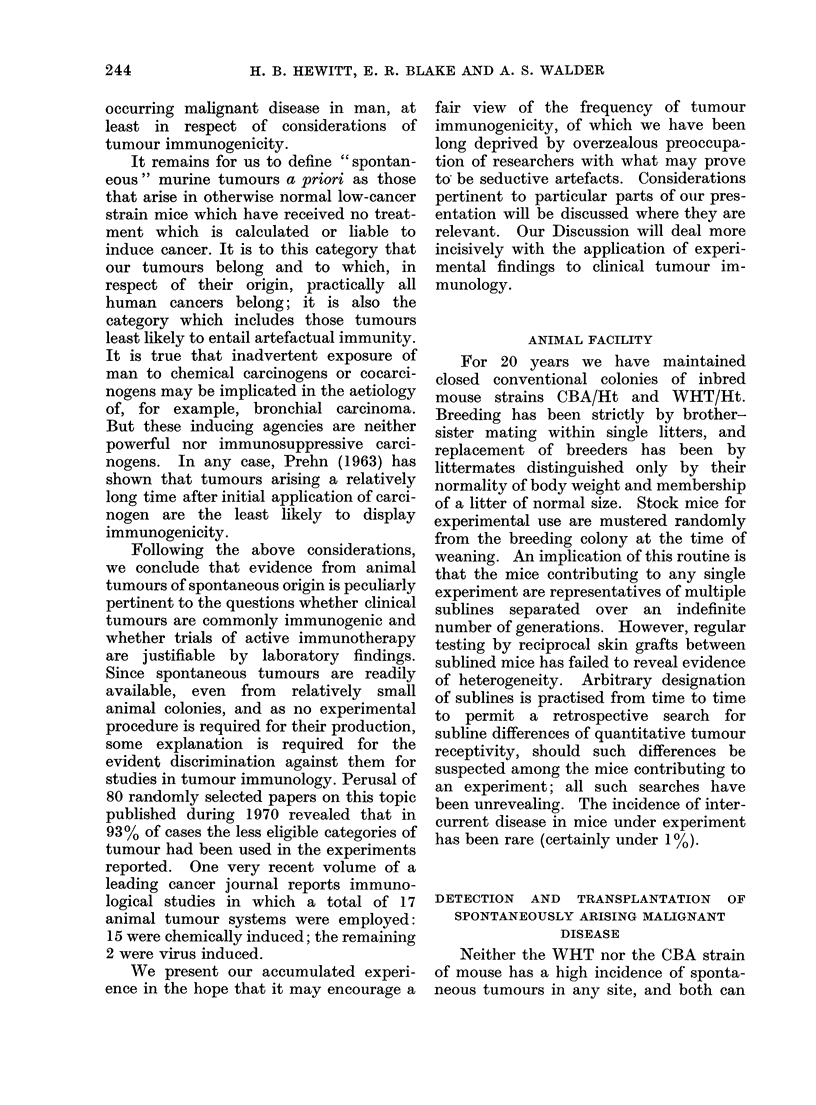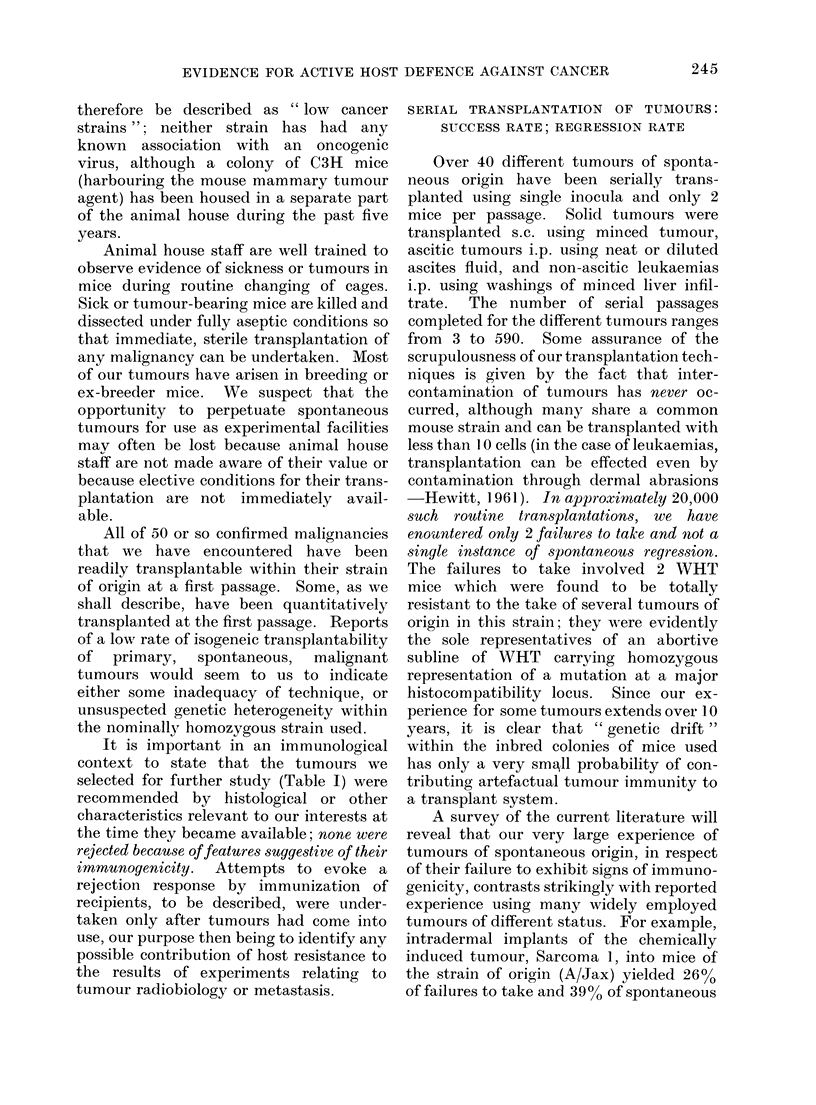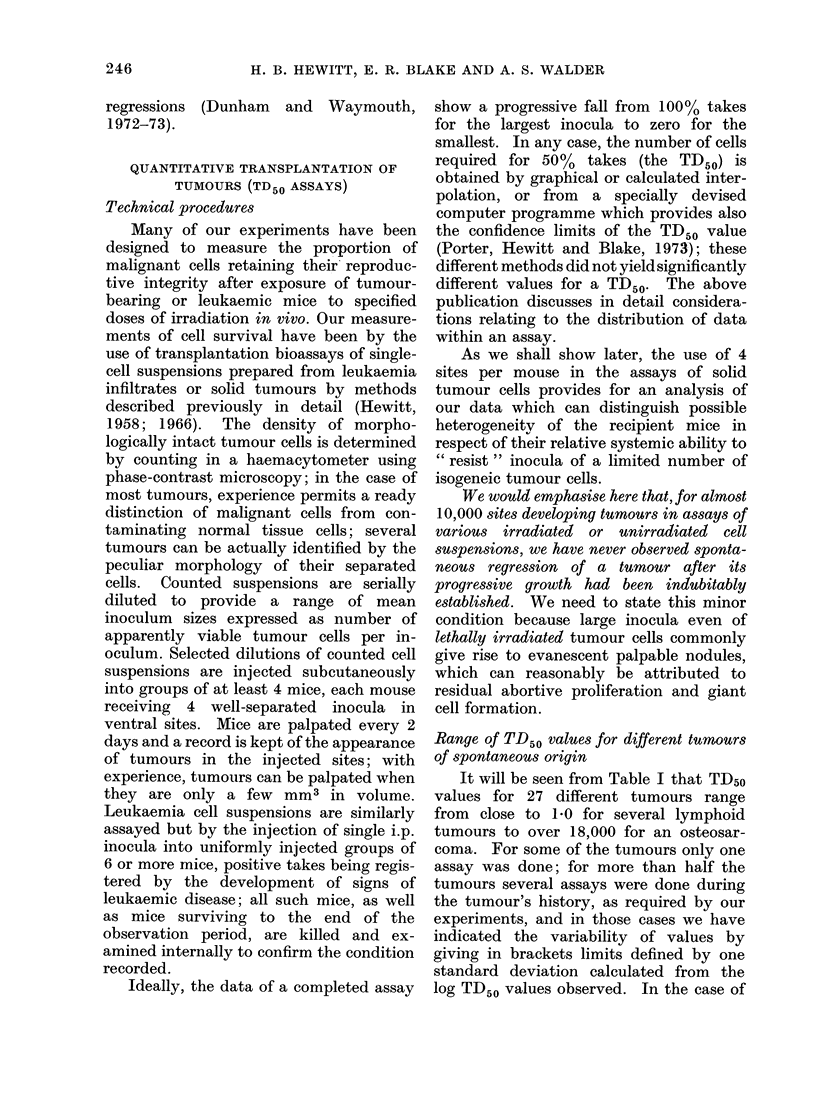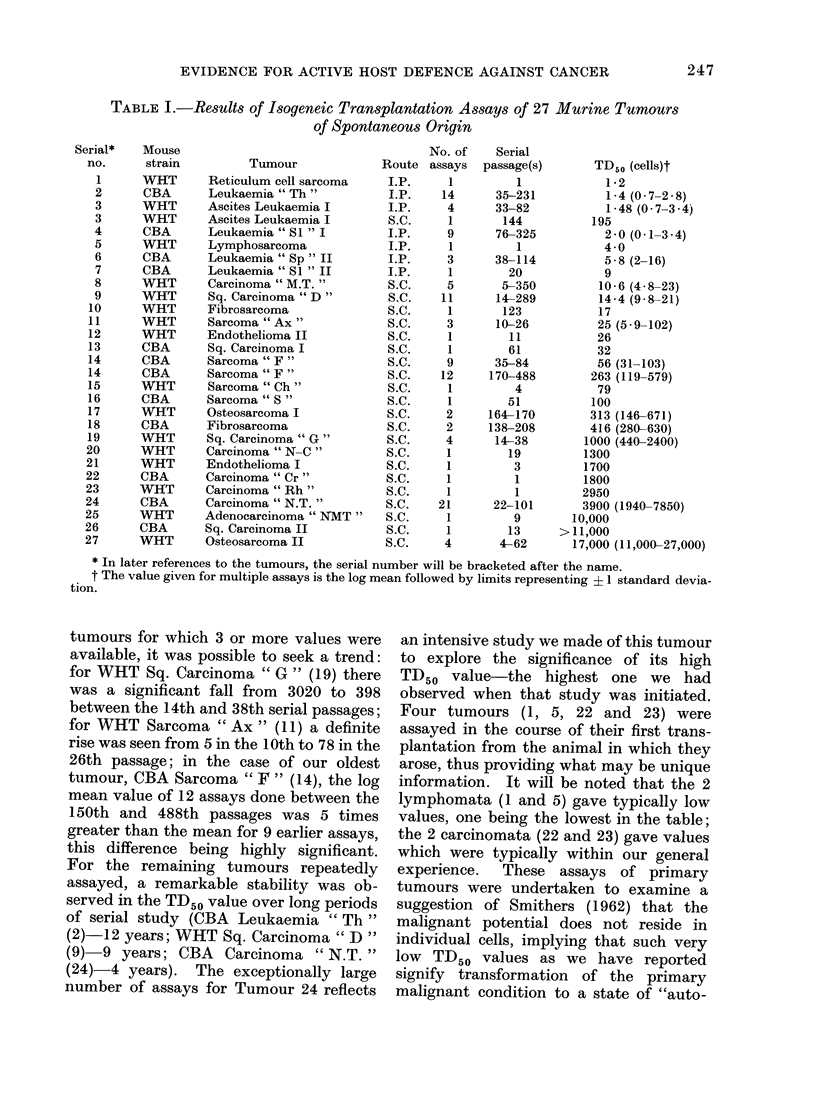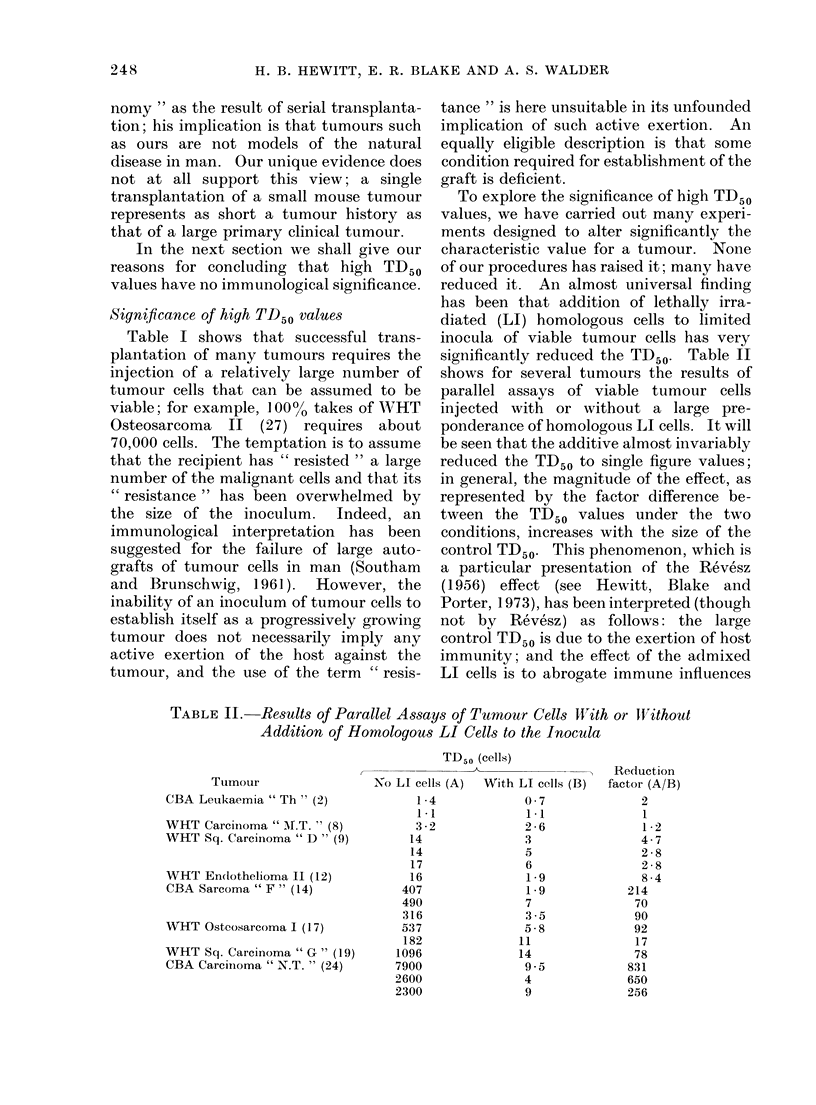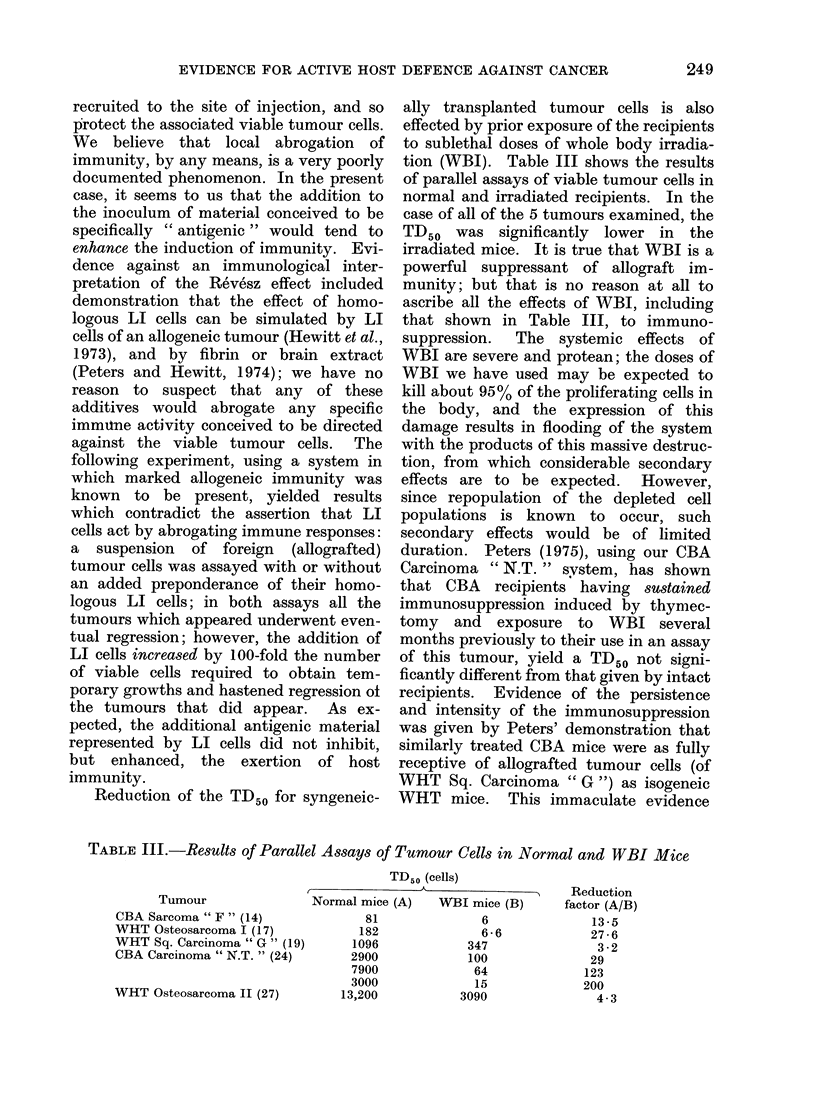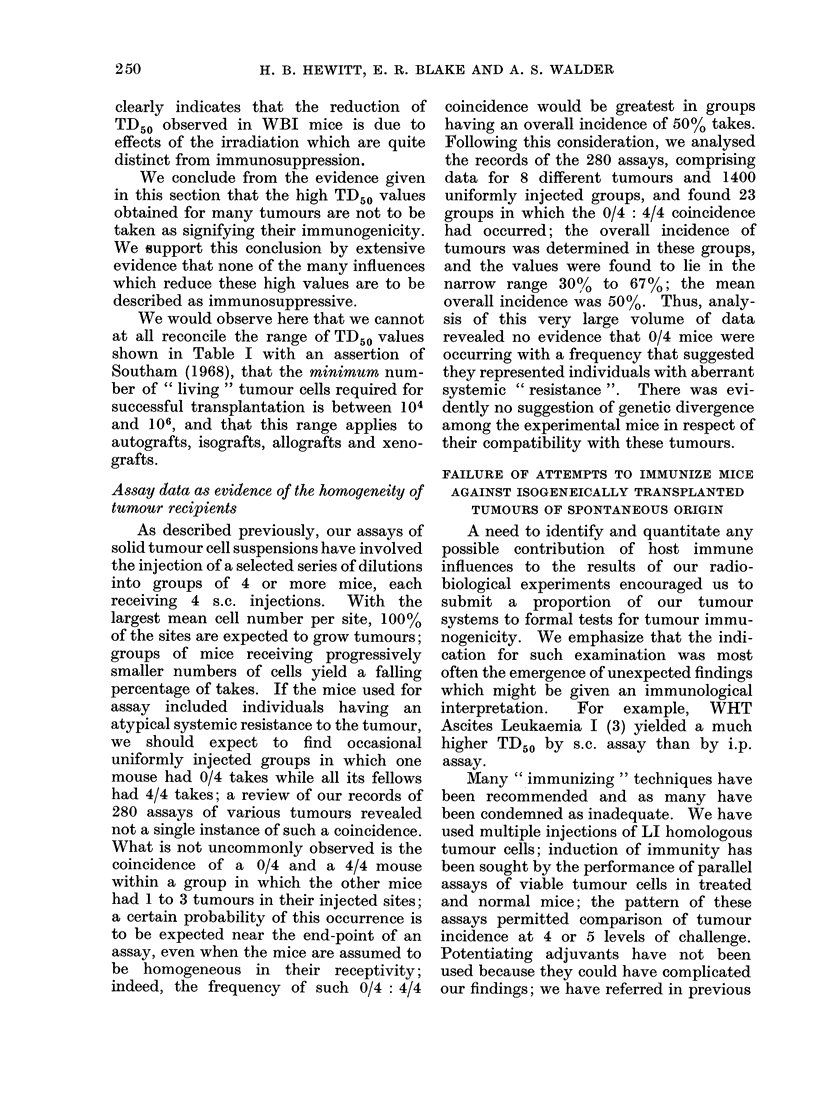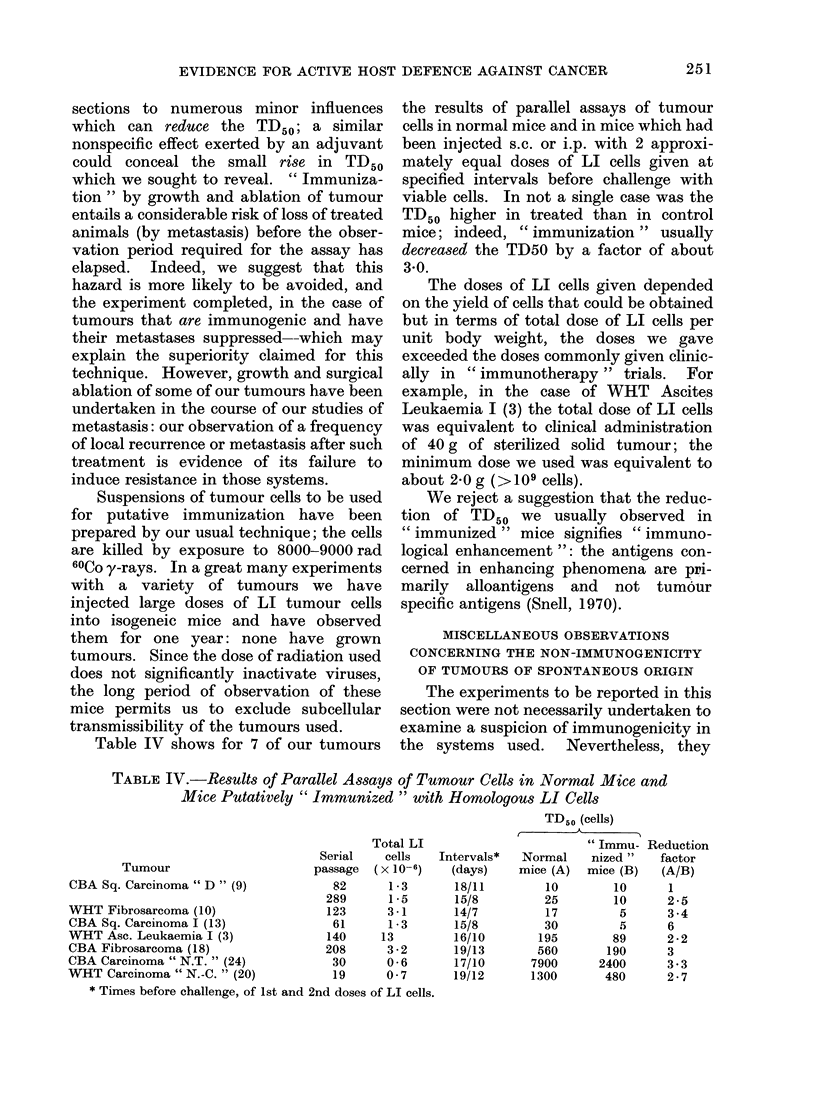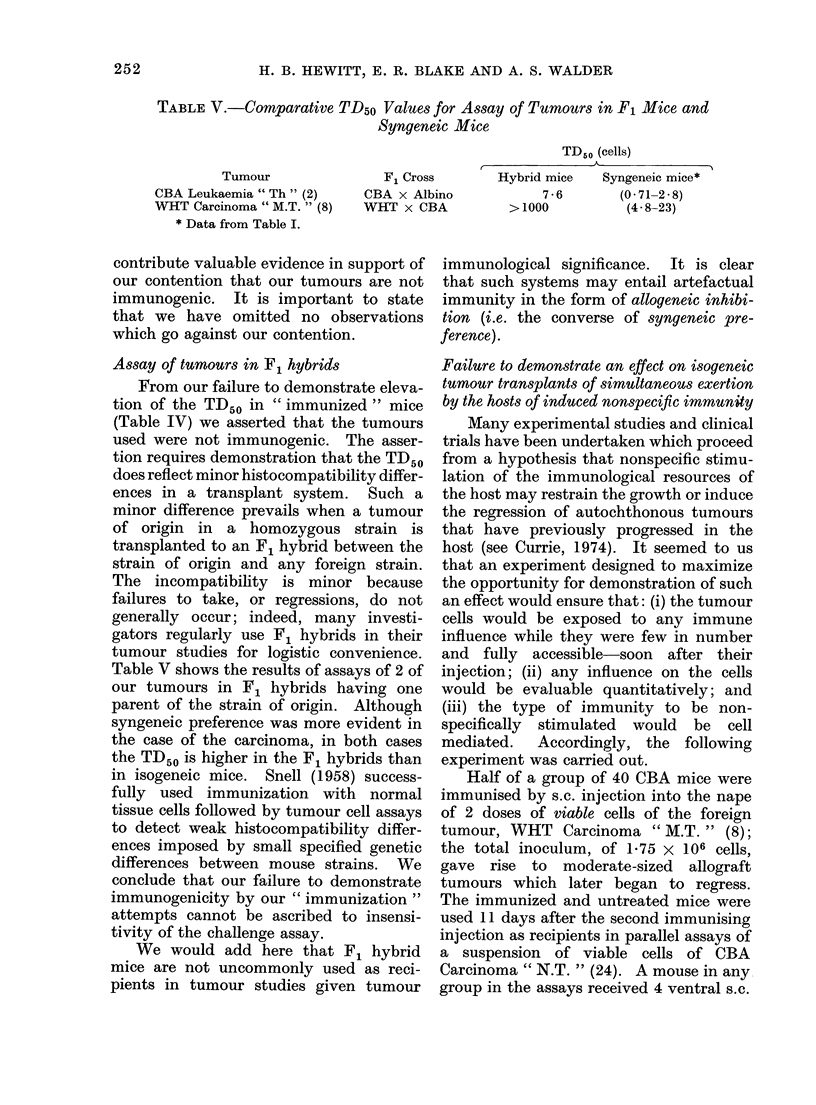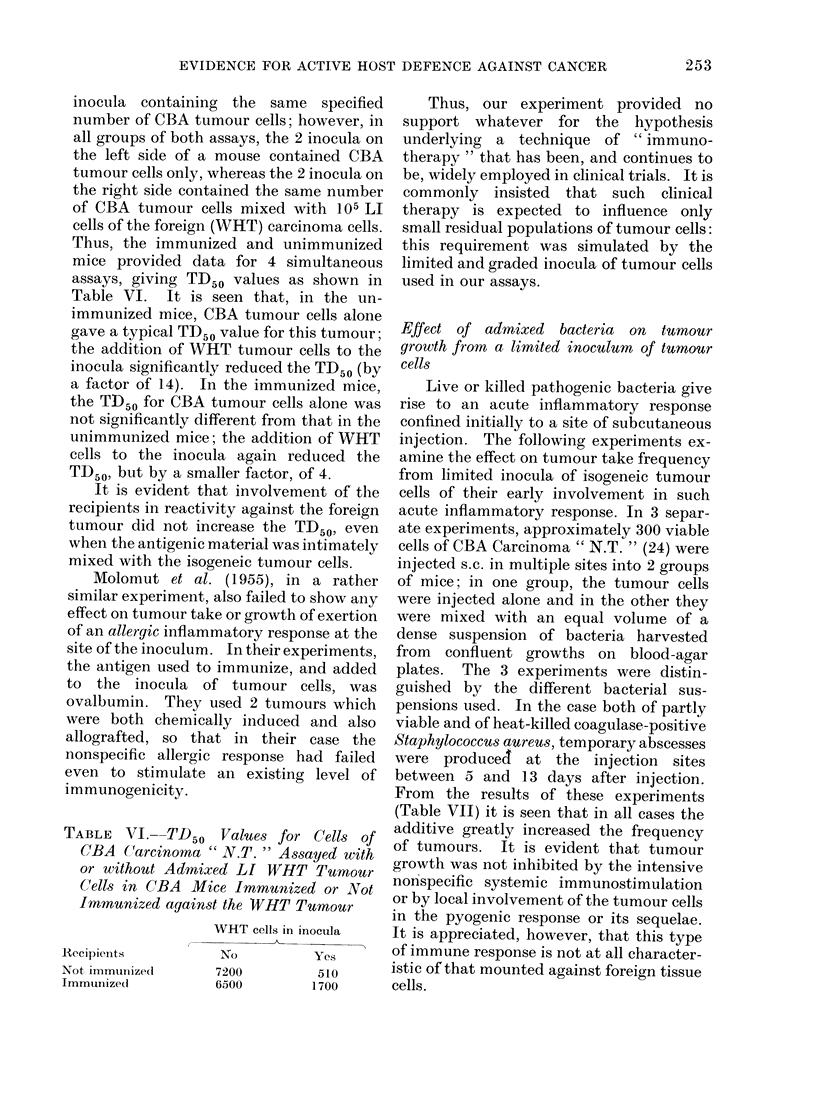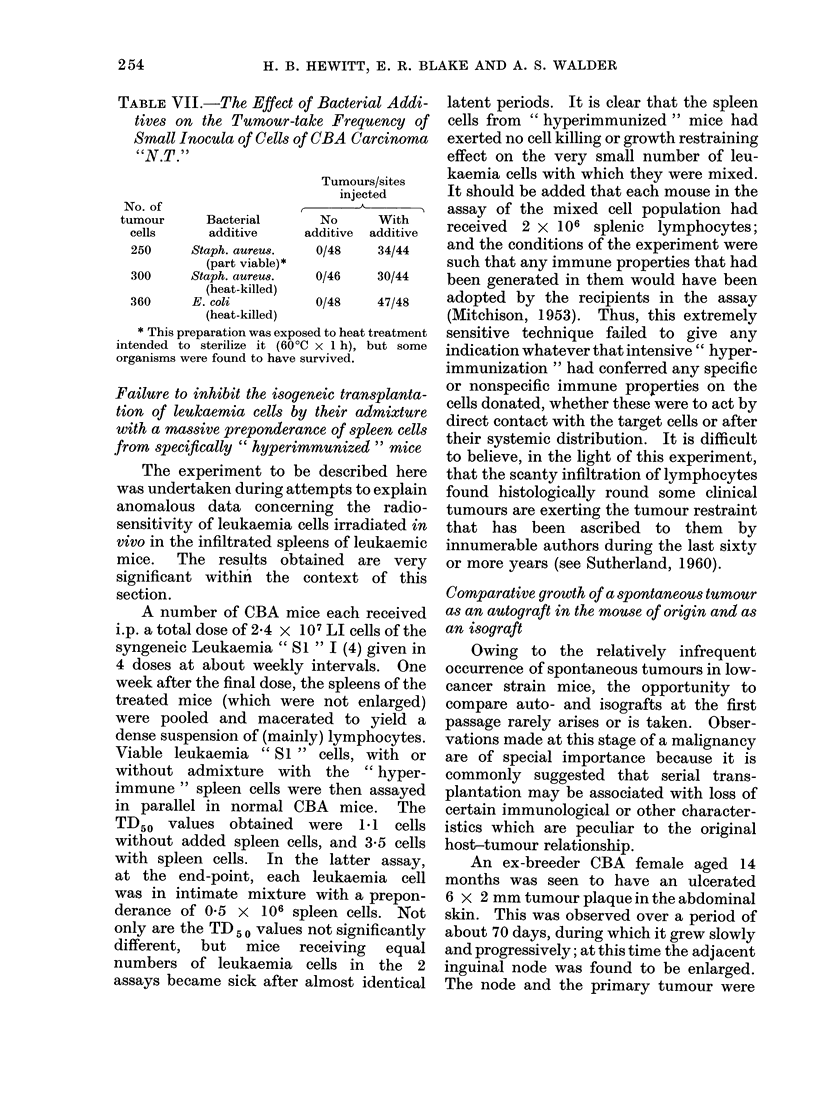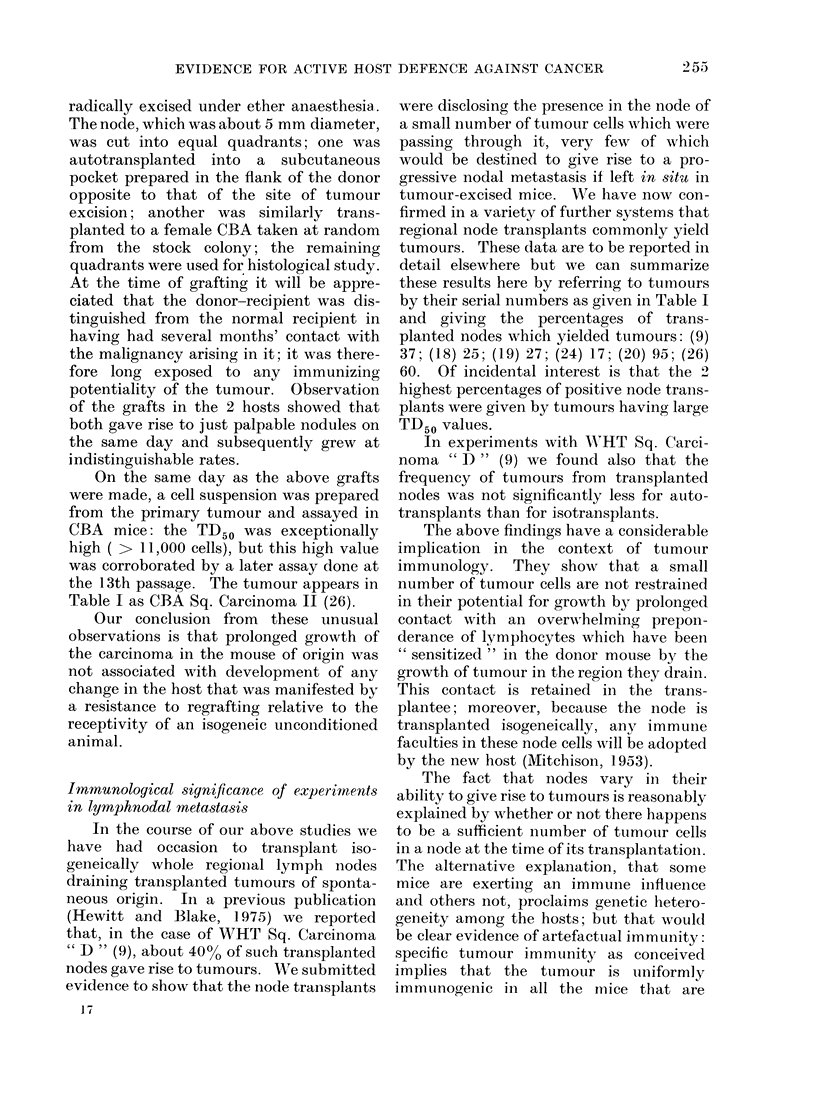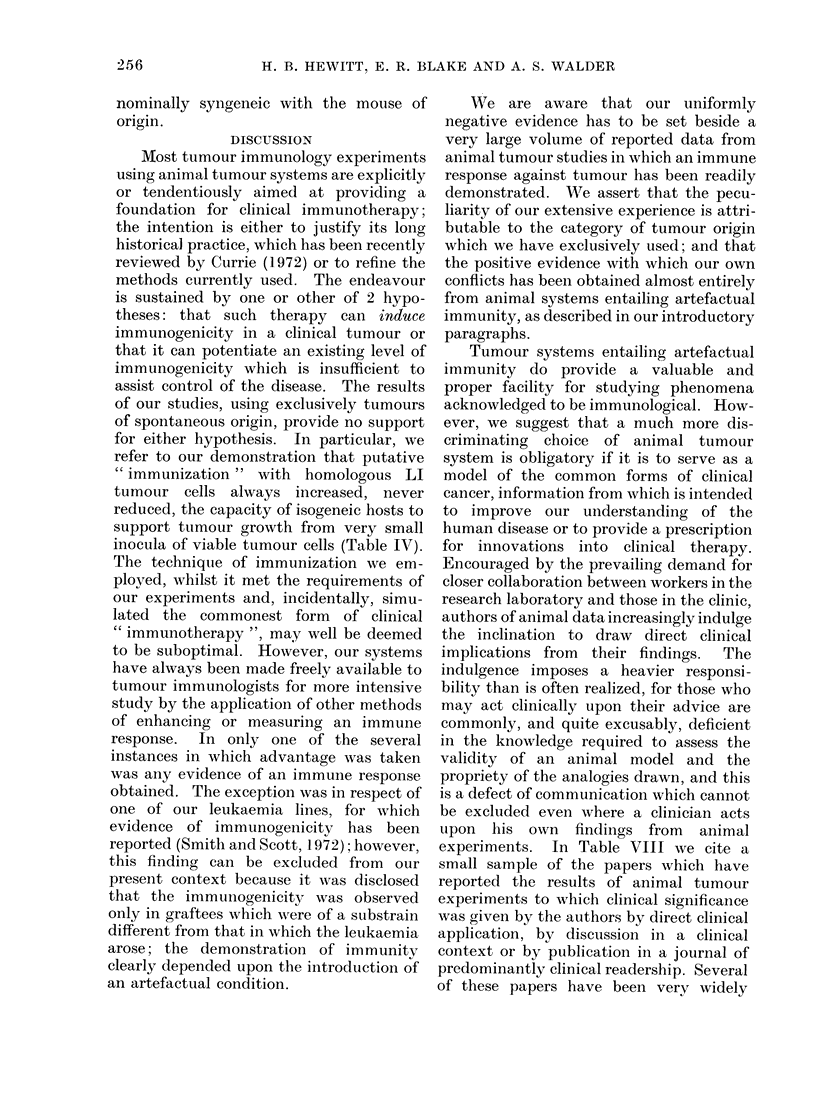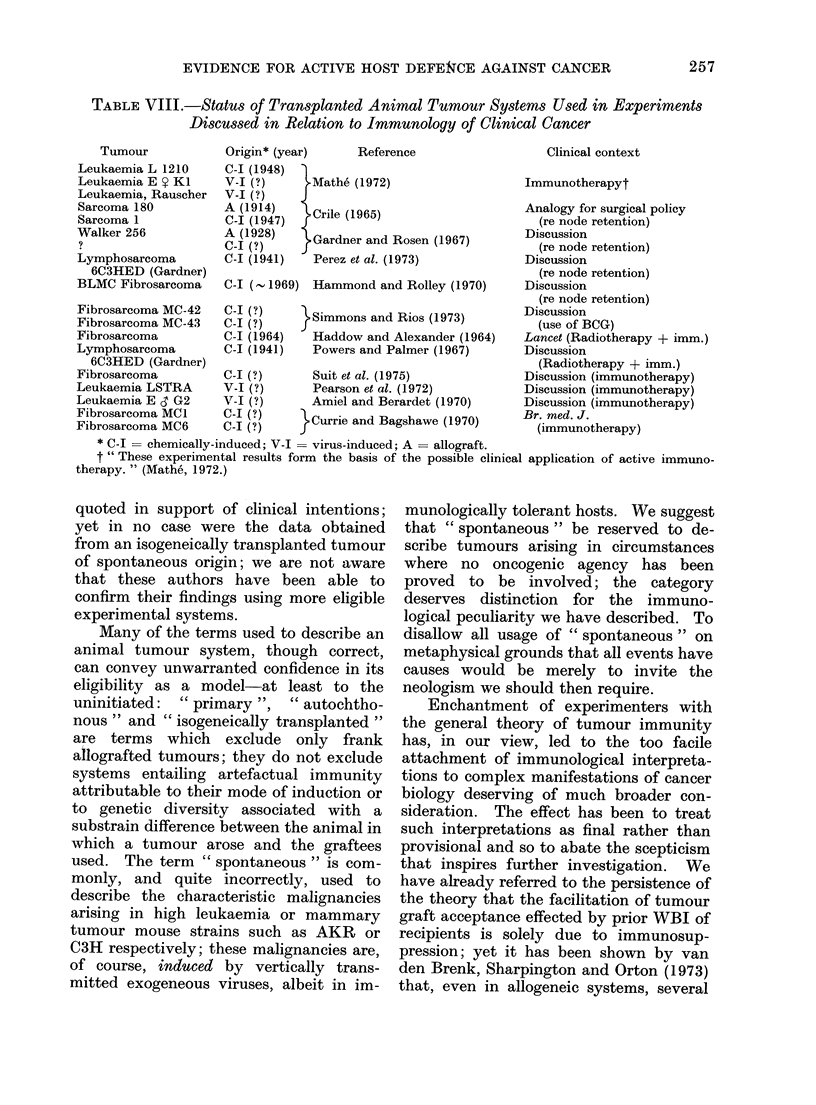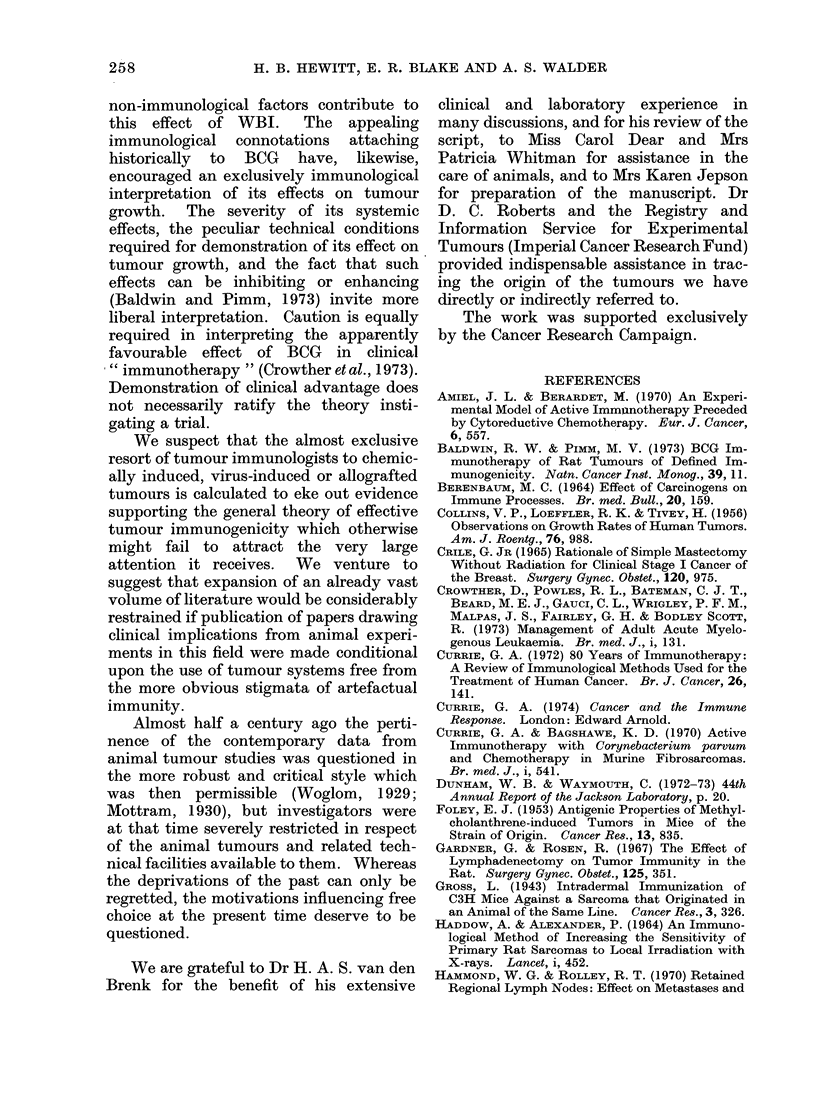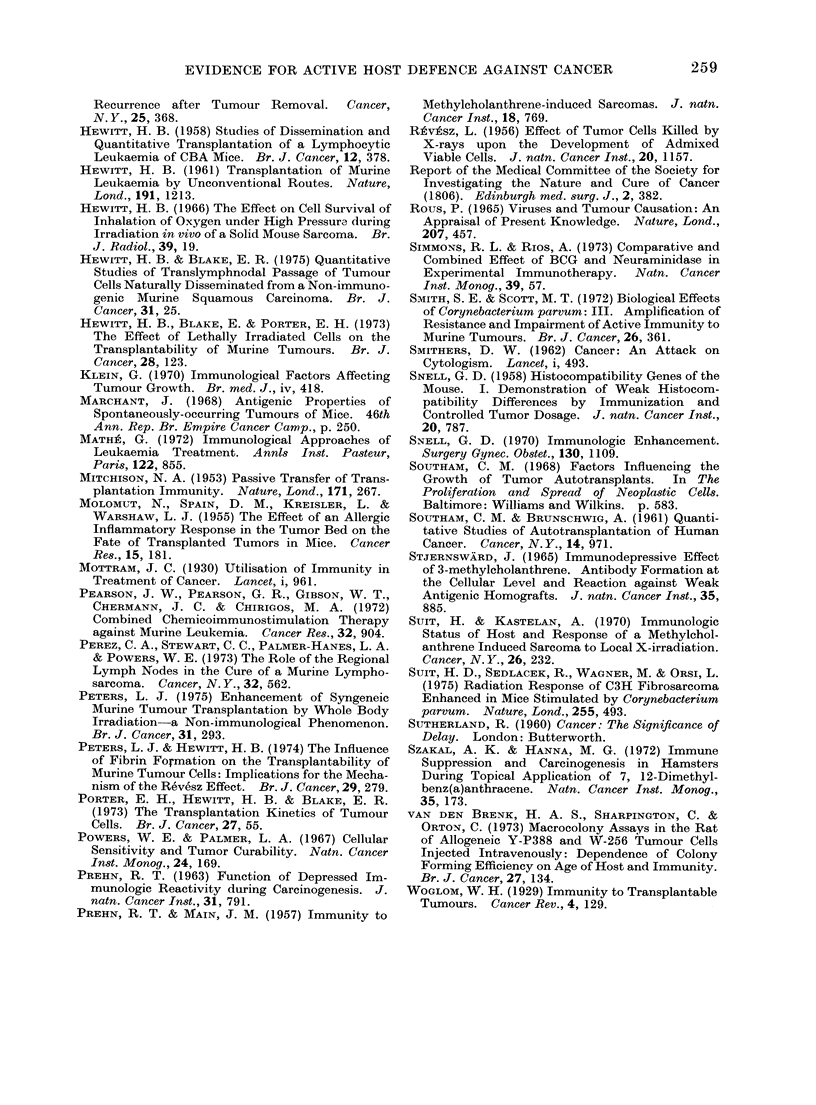# A critique of the evidence for active host defence against cancer, based on personal studies of 27 murine tumours of spontaneous origin.

**DOI:** 10.1038/bjc.1976.37

**Published:** 1976-03

**Authors:** H. B. Hewitt, E. R. Blake, A. S. Walder

## Abstract

Extensive experience with isotransplants of 27 different tumours (leukaemias, sarcomata, carcinomata), all of strictly spontaneous origin in laboratory bred mice of low cancer strains CBA/Ht and WHT/Ht, has revealed no evidence of tumour immunogenicity. Of approximately 20,000 maintenance transplants, none failed and none regressed; of almost 10,000 carefully observed tumours arising from small or minimal inocula of tumour cells, none spontaneously regressed. The number of injected viable tumour cells required to give a 50% probability of successful transplantation (the TD50) ranged from approximately 1 cell to greater than 10,000 cells among the 27 tumours; high TD50 values, which were dramatically reduced by various procedures having no immunological significance, did not signify active "resistance" of the hosts. In the case of all of 7 randomly selected tumours, prior "immunization" of recipients with homologous lethally irradiated cells increased their tumour receptivity. Several experiments using various tumours failed to give evidence that immunity could be non-specifically induced or that a massive preponderance of lymphocytes from specifically sensitized mice could inhibit tumour transplantation or growth in vivo; no trace of "resistance" to tumour was adopted by isogeneic recipients of lymphocytes from regional nodes of tumour bearers. A limited review of the recent literature on tumour immunity shows that practically all the animal data presented in support of a general theory of tumour immunogenicity or to provide a basis for active clinical immunotherapy have been obtained from transplanted tumour systems which entail artefactual immunity associated with viral or chemical induction of the tumours or their allogeneic transplantation. It is suggested that isotransplants of spontaneously arising tumours are the only appropriate models of human cancer and that any genuine rapport between the animal laboratory and the clinic requires their exclusive use.


*Br. J. Cancer* (1976) **33**, 241

### A CRITIQUE OF THE EVIDENCE FOR ACTIVE HOST DEFENCE AGAINST CANCER, BASED ON PERSONAL STUDIES OF 27 MURINE TUMOURS OF SPONTANEOUS ORIGIN

H. B. HEWITT, E. R. BLAKE AND A. S. WALDER

*From the C. R. C. Gray Laboratory, Mount Vernon Hospital, Northwood, Middlesex, HA6 2RN, England*

Received 3 October 1975 Accepted 29 November 1975

**Summary.—Extensive experience with isotransplants of 27 different tumours (leukaemias, sarcomata, carcinomata), all of strictly spontaneous origin in laboratory bred mice of low cancer strains CBA/Ht and WHT/Ht, has revealed no evidence of tumour immunogenicity. Of approximately 20,000 maintenance transplants, none failed and none regressed; of almost 10,000 carefully observed tumours arising from small or minimal inocula of tumour cells, none spontaneously regressed. The number of injected viable tumour cells required to give a 50% probability of successful transplantation (the $TD_{50}$) ranged from $\sim$1 cell to > 10,000 cells among the 27 tumours; high $TD_{50}$ values, which were dramatically reduced by various procedures having no immunological significance, did not signify active " resistance " of the hosts. In the case of all of 7 randomly selected tumours, prior " immunization " of recipients with homologous lethally irradiated cells *increased* their tumour receptivity.**

**Several experiments using various tumours failed to give evidence that immunity could be non-specifically induced or that a massive preponderance of lymphocytes from specifically sensitized mice could inhibit tumour transplantation or growth *in vivo*; no trace of " resistance " to tumour was adopted by isogeneic recipients of lymphocytes from regional nodes of tumour bearers. A limited review of the recent literature on tumour immunity shows that practically all the animal data presented in support of a general theory of tumour immunogenicity or to provide a basis for active clinical immunotherapy have been obtained from transplanted tumour systems which entail artefactual immunity associated with viral or chemical induction of the tumours or their allogeneic transplantation. It is suggested that isotransplants of spontaneously arising tumours are the only appropriate models of human cancer and that any genuine rapport between the animal laboratory and the clinic requires their exclusive use.**

WE REPORT here a considerable volume of data concerning the transplantation characteristics of a large number and variety of murine tumours having the distinction that they were all of strictly spontaneous origin in low cancer strain mice. The data, collected over many years of experimentation, have some additional claim to uniqueness from the exceptionally uniform conditions under which the information was obtained: the breeding colonies of inbred mice have been managed in every detail by one of us (A.S.W.); all other technical procedures have been carried out personally by the authors without additional assistance; to validate comparison of data between different tumours and from time to time over long periods of any one tumour's history, the routine technical procedures have not been varied in any significant particular; and, finally, a detailed record has been kept of the fate of every mouse used in every experiment, permitting the retrospective analysis of our experience which we are to report.

A feature of our experiments, required by the studies of tumour radiobiology and of metastasis to which they contributed, was that all transplantations (except maintenance and preparatory passages) were made quantitatively using counted tumour cell suspensions and mostly employing several uniformly injected sites per mouse. Since the success of such grafts is determined by some function relating the size of the inoculum and the receptivity of the injectee, it is clear that our collected data are in some way expressive of the host/tumour relationship; they deserve serious consideration in the context of tumour immunology. Whilst that topic has certainly not instigated our studies, the interpretation of our experimental results has required continual alertness to the possible complicity of an immunological response of the hosts. Our vigilance was stimulated by the prevalent theory that neoantigens necessarily emerge at malignant transformation, implying that potential tumour immunogenicity is intrinsic to tumour biology. From an early satisfaction that we had been fortunate to escape complications from this ingredient, we have progressed to our present conviction—that our experience conflicts absolutely with the broad assertion that tumour immunogenicity is a common and detectable feature of spontaneous cancer. We believe that this conflict arises out of our deliberate and exclusive use of tumours of spontaneous origin isogeneically transplanted within our own inbred mouse colonies.

Our purpose here is to present features of our broad experience which appear significant in the context of tumour immunology, as presented by the reports of host response against experimental tumours and in respect of its relevance to human cancer.

Consideration of our experience has compelled us to examine more closely the evidential basis of the concept of tumour immunogenicity as derived from clinical observation and, further, to examine critically the status of the profusion of animal tumour data that now underpins the fragile structure of clinical impression. Our object will be to enquire whether the apparent singularity of our findings can be ascribed to the category of tumour we have exclusively studied. We shall initiate this enquiry here and pursue it more pointedly in the Discussion of our findings.

A concept that the course of clinical cancer is the resultant of interaction between tumour growth potential and some active and inherent host defence against it has prevailed for over one and a half centuries (see Report, 1806). Perpetuation of the tradition is displayed in contemporary case reports, which very commonly attach immunological interpretations to unexpectedly favourable (or unfavourable) developments in the course of cancer, with careless disregard of the evidence required to validate such explanations for consoling (or disappointing) prognosis. A classic requirement for demonstration of immunity is some facility for detecting a differential response to sequential applications of an " antigen ". The facility is not provided by patients with established cancer, who have been in continuous contact with their cancers since their inception, usually many years previously (Collins, Loeffler and Tivey, 1956). It is clear that very subtle deviations from classic standards of evidence are required if effective immunological exertion is to be perceived in such circumstances. Indeed, it is significant that research in this field is currently sustained by a theory which entails the assumption of failure, relative or absolute, of an active host defence against progression of the disease which was never demonstrable.

The recent surge of publications on tumour immunity (typically represented in one leading cancer journal by an increase in the proportion of space devoted to them from 4% in 1966, to 13% in 1971, on to 27% in the first half of 1974) does not, in our view, substantiate any general theory of active defence against the clinical disease. *In vitro* demonstrations of tumour-specific or associated antigens or of various interactions between tumour cells, inflammatory cells and humoral factors, do not constitute evidence of effective *immunogenicity*; it has yet to be shown that they are more than epiphenomena or that they betoken *in vivo* influences effective in restraining the disease (Currie, 1974).

Animal tumour models are certainly required if evidence for induced resistance against cancer is to be provided in a form that is consistent with classic immunological precepts; that is, a differential response to challenge with viable cancer cells has to be demonstrated between normal animals and those that have been conditioned by contact with specific challenge material. There is no doubt that induction of immunity against tumours has been so demonstrated with undiminished frequency from the beginning of experimental cancer research. The telling question is whether the transplanted tumour systems that have been and are being used are valid models of the natural disease; they must certainly be disqualified from this role if the immune reactivity displayed can be reasonably attributed to one or other of a number of laboratory artefacts that can be inferred from a tumour's origin or conditions of transplantation. Since the simultaneous exertion of natural *and* artefactual tumour immunity presents the daunting problem of their distinction and separate measurement, the ineligibility of a system has to be pronounced from circumstantial evidence alone.

The status of the systems we have used must be considered in relation to the several categories into which animal tumour systems can be classified:

1. Allografted tumours.
2. Virus-induced tumours.
3. Chemically induced tumours.
4. Spontaneous tumours.

All allografted tumours entail artefactual immunity; they include not only old tumours of unspecified genetic origin but also systems in which transplantation is nominally isogeneic, but in which the long history of a tumour and particularly its origin in some other laboratory, make it highly probable that genetic divergence has taken place between the recipients used and the substrain of origin; even transplants into $F_1$ hybrid mice are not strictly isogeneic. In the case of tumours of categories 2–4, our discussion of their eligibility as models will assume strictly isogeneic transplantation. Tumours induced by a specified virus are well known to share a membrane antigen and a vast literature testifies to their, often strong, immunogenicity. What we must acknowledge here is that virus-induced tumours are rarities and that there is no reason to suppose that the generality of tumours are so induced (Rous, 1965); certainly no human malignancy, with the possibly impending exception of Burkitt's disease, has been proved to be of viral causation.

Chemically induced (C–I) tumours are usually immunogenic; indeed, the earliest reports of this peculiarity (Gross, 1943; Foley, 1953; Prehn and Main, 1957) presented the finding as a discovery. Although a special status for C-I tumours on this account is commonly refused, all comparisons that have been made have revealed a degree of immunogenicity in C-I tumours which was rarely, if ever, encountered in tumours of spontaneous origin (Prehn and Main, 1957; Marchant, 1968; Klein, 1970; Suit and Kastelan, 1970). The immunogenicity of C-I tumours is associated with the immunosuppressive activity of the powerful carcinogenic agents almost invariably used in the laboratory for tumour induction (Prehn, 1963; Stjernswärd, 1965; Berenbaum, 1964, Szakal and Hanna, 1972). Prehn (1963) considered that the immunosuppressive action of chemical carcinogens might well be an essential component of the mechanism of their carcinogenicity. The above abbreviated review is, we believe, sufficient to deny C-I tumours any status as acceptable models of naturally occurring malignant disease in man, at least in respect of considerations of tumour immunogenicity.

It remains for us to define "spontaneous" murine tumours *a priori* as those that arise in otherwise normal low-cancer strain mice which have received no treatment which is calculated or liable to induce cancer. It is to this category that our tumours belong and to which, in respect of their origin, practically all human cancers belong; it is also the category which includes those tumours least likely to entail artefactual immunity. It is true that inadvertent exposure of man to chemical carcinogens or cocarcinogens may be implicated in the aetiology of, for example, bronchial carcinoma. But these inducing agencies are neither powerful nor immunosuppressive carcinogens. In any case, Prehn (1963) has shown that tumours arising a relatively long time after initial application of carcinogen are the least likely to display immunogenicity.

Following the above considerations, we conclude that evidence from animal tumours of spontaneous origin is peculiarly pertinent to the questions whether clinical tumours are commonly immunogenic and whether trials of active immunotherapy are justifiable by laboratory findings. Since spontaneous tumours are readily available, even from relatively small animal colonies, and as no experimental procedure is required for their production, some explanation is required for the evident discrimination against them for studies in tumour immunology. Perusal of 80 randomly selected papers on this topic published during 1970 revealed that in 93% of cases the less eligible categories of tumour had been used in the experiments reported. One very recent volume of a leading cancer journal reports immunological studies in which a total of 17 animal tumour systems were employed: 15 were chemically induced; the remaining 2 were virus induced.

We present our accumulated experience in the hope that it may encourage a fair view of the frequency of tumour immunogenicity, of which we have been long deprived by overzealous preoccupation of researchers with what may prove to be seductive artefacts. Considerations pertinent to particular parts of our presentation will be discussed where they are relevant. Our Discussion will deal more incisively with the application of experimental findings to clinical tumour immunology.

### ANIMAL FACILITY

For 20 years we have maintained closed conventional colonies of inbred mouse strains CBA/Ht and WHT/Ht. Breeding has been strictly by brother–sister mating within single litters, and replacement of breeders has been by littermates distinguished only by their normality of body weight and membership of a litter of normal size. Stock mice for experimental use are mustered randomly from the breeding colony at the time of weaning. An implication of this routine is that the mice contributing to any single experiment are representatives of multiple sublines separated over an indefinite number of generations. However, regular testing by reciprocal skin grafts between sublined mice has failed to reveal evidence of heterogeneity. Arbitrary designation of sublines is practised from time to time to permit a retrospective search for subline differences of quantitative tumour receptivity, should such differences be suspected among the mice contributing to an experiment; all such searches have been unrevealing. The incidence of intercurrent disease in mice under experiment has been rare (certainly under 1%).

### DETECTION AND TRANSPLANTATION OF SPONTANEOUSLY ARISING MALIGNANT DISEASE

Neither the WHT nor the CBA strain of mouse has a high incidence of spontaneous tumours in any site, and both can therefore be described as " low cancer strains "; neither strain has had any known association with an oncogenic virus, although a colony of C3H mice (harbouring the mouse mammary tumour agent) has been housed in a separate part of the animal house during the past five years.

Animal house staff are well trained to observe evidence of sickness or tumours in mice during routine changing of cages. Sick or tumour-bearing mice are killed and dissected under fully aseptic conditions so that immediate, sterile transplantation of any malignancy can be undertaken. Most of our tumours have arisen in breeding or ex-breeder mice. We suspect that the opportunity to perpetuate spontaneous tumours for use as experimental facilities may often be lost because animal house staff are not made aware of their value or because elective conditions for their transplantation are not immediately available.

All of 50 or so confirmed malignancies that we have encountered have been readily transplantable within their strain of origin at a first passage. Some, as we shall describe, have been quantitatively transplanted at the first passage. Reports of a low rate of isogeneic transplantability of primary, spontaneous, malignant tumours would seem to us to indicate either some inadequacy of technique, or unsuspected genetic heterogeneity within the nominally homozygous strain used.

It is important in an immunological context to state that the tumours we selected for further study (Table I) were recommended by histological or other characteristics relevant to our interests at the time they became available; *none were rejected because of features suggestive of their immunogenicity.* Attempts to evoke a rejection response by immunization of recipients, to be described, were undertaken only after tumours had come into use, our purpose then being to identify any possible contribution of host resistance to the results of experiments relating to tumour radiobiology or metastasis.

SERIAL TRANSPLANTATION OF TUMOURS: SUCCESS RATE; REGRESSION RATE

Over 40 different tumours of spontaneous origin have been serially transplanted using single inocula and only 2 mice per passage. Solid tumours were transplanted s.c. using minced tumour, ascitic tumours i.p. using neat or diluted ascites fluid, and non-ascitic leukaemias i.p. using washings of minced liver infiltrate. The number of serial passages completed for the different tumours ranges from 3 to 590. Some assurance of the scrupulousness of our transplantation techniques is given by the fact that intercontamination of tumours has *never* occurred, although many share a common mouse strain and can be transplanted with less than 10 cells (in the case of leukaemias, transplantation can be effected even by contamination through dermal abrasions —Hewitt, 1961). *In approximately* 20,000 *such routine transplantations, we have enountered only* 2 *failures to take and not a single instance of spontaneous regression.* The failures to take involved 2 WHT mice which were found to be totally resistant to the take of several tumours of origin in this strain; they were evidently the sole representatives of an abortive subline of WHT carrying homozygous representation of a mutation at a major histocompatibility locus. Since our experience for some tumours extends over 10 years, it is clear that " genetic drift " within the inbred colonies of mice used has only a very small probability of contributing artefactual tumour immunity to a transplant system.

A survey of the current literature will reveal that our very large experience of tumours of spontaneous origin, in respect of their failure to exhibit signs of immunogenicity, contrasts strikingly with reported experience using many widely employed tumours of different status. For example, intradermal implants of the chemically induced tumour, Sarcoma 1, into mice of the strain of origin (A/Jax) yielded 26% of failures to take and 39% of spontaneous regressions (Dunham and Waymouth, 1972–73).

### QUANTITATIVE TRANSPLANTATION OF TUMOURS ($TD_{50}$ ASSAYS)

*Technical procedures*

Many of our experiments have been designed to measure the proportion of malignant cells retaining their reproductive integrity after exposure of tumour-bearing or leukaemic mice to specified doses of irradiation *in vivo*. Our measurements of cell survival have been by the use of transplantation bioassays of single-cell suspensions prepared from leukaemia infiltrates or solid tumours by methods described previously in detail (Hewitt, 1958; 1966). The density of morphologically intact tumour cells is determined by counting in a haemacytometer using phase-contrast microscopy; in the case of most tumours, experience permits a ready distinction of malignant cells from contaminating normal tissue cells; several tumours can be actually identified by the peculiar morphology of their separated cells. Counted suspensions are serially diluted to provide a range of mean inoculum sizes expressed as number of apparently viable tumour cells per inoculum. Selected dilutions of counted cell suspensions are injected subcutaneously into groups of at least 4 mice, each mouse receiving 4 well-separated inocula in ventral sites. Mice are palpated every 2 days and a record is kept of the appearance of tumours in the injected sites; with experience, tumours can be palpated when they are only a few $mm^3$ in volume. Leukaemia cell suspensions are similarly assayed but by the injection of single i.p. inocula into uniformly injected groups of 6 or more mice, positive takes being registered by the development of signs of leukaemic disease; all such mice, as well as mice surviving to the end of the observation period, are killed and examined internally to confirm the condition recorded.

Ideally, the data of a completed assay show a progressive fall from 100% takes for the largest inocula to zero for the smallest. In any case, the number of cells required for 50% takes (the $TD_{50}$) is obtained by graphical or calculated interpolation, or from a specially devised computer programme which provides also the confidence limits of the $TD_{50}$ value (Porter, Hewitt and Blake, 1973); these different methods did not yield significantly different values for a $TD_{50}$. The above publication discusses in detail considerations relating to the distribution of data within an assay.

As we shall show later, the use of 4 sites per mouse in the assays of solid tumour cells provides for an analysis of our data which can distinguish possible heterogeneity of the recipient mice in respect of their relative systemic ability to " resist " inocula of a limited number of isogeneic tumour cells.

*We would emphasise here that, for almost* 10,000 *sites developing tumours in assays of various irradiated or unirradiated cell suspensions, we have never observed spontaneous regression of a tumour after its progressive growth had been indubitably established.* We need to state this minor condition because large inocula even of *lethally irradiated* tumour cells commonly give rise to evanescent palpable nodules, which can reasonably be attributed to residual abortive proliferation and giant cell formation.

*Range of $TD_{50}$ values for different tumours of spontaneous origin*

It will be seen from Table I that $TD_{50}$ values for 27 different tumours range from close to 1·0 for several lymphoid tumours to over 18,000 for an osteosarcoma. For some of the tumours only one assay was done; for more than half the tumours several assays were done during the tumour's history, as required by our experiments, and in those cases we have indicated the variability of values by giving in brackets limits defined by one standard deviation calculated from the log $TD_{50}$ values observed. In the case of

TABLE I.—*Results of Isogeneic Transplantation Assays of 27 Murine Tumours of Spontaneous Origin*

| Serial* no. | Mouse strain | Tumour | Route | No. of assays | Serial passage(s) | $TD_{50}$ (cells)† |
|---|---|---|---|---|---|---|
| 1 | WHT | Reticulum cell sarcoma | I.P. | 1 | 1 | 1·2 |
| 2 | CBA | Leukaemia " Th " | I.P. | 14 | 35–231 | 1·4 (0·7–2·8) |
| 3 | WHT | Ascites Leukaemia I | I.P. | 4 | 33–82 | 1·48 (0·7–3·4) |
| 3 | WHT | Ascites Leukaemia I | S.C. | 1 | 144 | 195 |
| 4 | CBA | Leukaemia " Sl " I | I.P. | 9 | 76–325 | 2·0 (0·1–3·4) |
| 5 | WHT | Lymphosarcoma | I.P. | 1 | 1 | 4·0 |
| 6 | CBA | Leukaemia " Sp " II | I.P. | 3 | 38–114 | 5·8 (2–16) |
| 7 | CBA | Leukaemia " Sl " II | I.P. | 1 | 20 | 9 |
| 8 | WHT | Carcinoma " M.T. " | S.C. | 5 | 5–350 | 10·6 (4·8–23) |
| 9 | WHT | Sq. Carcinoma " D " | S.C. | 11 | 14–289 | 14·4 (9·8–21) |
| 10 | WHT | Fibrosarcoma | S.C. | 1 | 123 | 17 |
| 11 | WHT | Sarcoma " Ax " | S.C. | 3 | 10–26 | 25 (5·9–102) |
| 12 | WHT | Endothelioma II | S.C. | 1 | 11 | 26 |
| 13 | CBA | Sq. Carcinoma I | S.C. | 1 | 61 | 32 |
| 14 | CBA | Sarcoma " F " | S.C. | 9 | 35–84 | 56 (31–103) |
| 14 | CBA | Sarcoma " F " | S.C. | 12 | 170–488 | 263 (119–579) |
| 15 | WHT | Sarcoma " Ch " | S.C. | 1 | 4 | 79 |
| 16 | CBA | Sarcoma " S " | S.C. | 1 | 51 | 100 |
| 17 | WHT | Osteosarcoma I | S.C. | 2 | 164–170 | 313 (146–671) |
| 18 | CBA | Fibrosarcoma | S.C. | 2 | 138–208 | 416 (280–630) |
| 19 | WHT | Sq. Carcinoma " G " | S.C. | 4 | 14–38 | 1000 (440–2400) |
| 20 | WHT | Carcinoma " N–C " | S.C. | 1 | 19 | 1300 |
| 21 | WHT | Endothelioma I | S.C. | 1 | 3 | 1700 |
| 22 | CBA | Carcinoma " Cr " | S.C. | 1 | 1 | 1800 |
| 23 | WHT | Carcinoma " Rh " | S.C. | 1 | 1 | 2950 |
| 24 | CBA | Carcinoma " N.T. " | S.C. | 21 | 22–101 | 3900 (1940–7850) |
| 25 | WHT | Adenocarcinoma " NMT " | S.C. | 1 | 9 | 10,000 |
| 26 | CBA | Sq. Carcinoma II | S.C. | 1 | 13 | >11,000 |
| 27 | WHT | Osteosarcoma II | S.C. | 4 | 4–62 | 17,000 (11,000–27,000) |

* In later references to the tumours, the serial number will be bracketed after the name.

† The value given for multiple assays is the log mean followed by limits representing $\pm 1$ standard deviation.

tumours for which 3 or more values were available, it was possible to seek a trend: for WHT Sq. Carcinoma " G " (19) there was a significant fall from 3020 to 398 between the 14th and 38th serial passages; for WHT Sarcoma " Ax " (11) a definite rise was seen from 5 in the 10th to 78 in the 26th passage; in the case of our oldest tumour, CBA Sarcoma " F " (14), the log mean value of 12 assays done between the 150th and 488th passages was 5 times greater than the mean for 9 earlier assays, this difference being highly significant. For the remaining tumours repeatedly assayed, a remarkable stability was observed in the $TD_{50}$ value over long periods of serial study (CBA Leukaemia " Th " (2)—12 years; WHT Sq. Carcinoma " D " (9)—9 years; CBA Carcinoma " N.T. " (24)—4 years). The exceptionally large number of assays for Tumour 24 reflects an intensive study we made of this tumour to explore the significance of its high $TD_{50}$ value—the highest one we had observed when that study was initiated. Four tumours (1, 5, 22 and 23) were assayed in the course of their first transplantation from the animal in which they arose, thus providing what may be unique information. It will be noted that the 2 lymphomata (1 and 5) gave typically low values, one being the lowest in the table; the 2 carcinomata (22 and 23) gave values which were typically within our general experience. These assays of primary tumours were undertaken to examine a suggestion of Smithers (1962) that the malignant potential does not reside in individual cells, implying that such very low $TD_{50}$ values as we have reported signify transformation of the primary malignant condition to a state of "autonomy " as the result of serial transplantation; his implication is that tumours such as ours are not models of the natural disease in man. Our unique evidence does not at all support this view; a single transplantation of a small mouse tumour represents as short a tumour history as that of a large primary clinical tumour.

In the next section we shall give our reasons for concluding that high $TD_{50}$ values have no immunological significance.

*Significance of high $TD_{50}$ values*

Table I shows that successful transplantation of many tumours requires the injection of a relatively large number of tumour cells that can be assumed to be viable; for example, 100% takes of WHT Osteosarcoma II (27) requires about 70,000 cells. The temptation is to assume that the recipient has " resisted " a large number of the malignant cells and that its " resistance " has been overwhelmed by the size of the inoculum. Indeed, an immunological interpretation has been suggested for the failure of large autografts of tumour cells in man (Southam and Brunschwig, 1961). However, the inability of an inoculum of tumour cells to establish itself as a progressively growing tumour does not necessarily imply any active exertion of the host against the tumour, and the use of the term " resistance " is here unsuitable in its unfounded implication of such active exertion. An equally eligible description is that some condition required for establishment of the graft is deficient.

To explore the significance of high $TD_{50}$ values, we have carried out many experiments designed to alter significantly the characteristic value for a tumour. None of our procedures has raised it; many have reduced it. An almost universal finding has been that addition of lethally irradiated (LI) homologous cells to limited inocula of viable tumour cells has very significantly reduced the $TD_{50}$. Table II shows for several tumours the results of parallel assays of viable tumour cells injected with or without a large preponderance of homologous LI cells. It will be seen that the additive almost invariably reduced the $TD_{50}$ to single figure values; in general, the magnitude of the effect, as represented by the factor difference between the $TD_{50}$ values under the two conditions, increases with the size of the control $TD_{50}$. This phenomenon, which is a particular presentation of the Révész (1956) effect (see Hewitt, Blake and Porter, 1973), has been interpreted (though not by Révész) as follows: the large control $TD_{50}$ is due to the exertion of host immunity; and the effect of the admixed LI cells is to abrogate immune influences

TABLE II.—*Results of Parallel Assays of Tumour Cells With or Without Addition of Homologous LI Cells to the Inocula*

| Tumour | $TD_{50}$ (cells) No LI cells (A) | $TD_{50}$ (cells) With LI cells (B) | Reduction factor (A/B) |
|---|---|---|---|
| CBA Leukaemia " Th " (2) | 1·4 | 0·7 | 2 |
| | 1·1 | 1·1 | 1 |
| WHT Carcinoma " M.T. " (8) | 3·2 | 2·6 | 1·2 |
| WHT Sq. Carcinoma " D " (9) | 14 | 3 | 4·7 |
| | 14 | 5 | 2·8 |
| | 17 | 6 | 2·8 |
| WHT Endothelioma II (12) | 16 | 1·9 | 8·4 |
| CBA Sarcoma " F " (14) | 407 | 1·9 | 214 |
| | 490 | 7 | 70 |
| | 316 | 3·5 | 90 |
| WHT Osteosarcoma I (17) | 537 | 5·8 | 92 |
| | 182 | 11 | 17 |
| WHT Sq. Carcinoma " G " (19) | 1096 | 14 | 78 |
| CBA Carcinoma " N.T. " (24) | 7900 | 9·5 | 831 |
| | 2600 | 4 | 650 |
| | 2300 | 9 | 256 |

recruited to the site of injection, and so protect the associated viable tumour cells. We believe that local abrogation of immunity, by any means, is a very poorly documented phenomenon. In the present case, it seems to us that the addition to the inoculum of material conceived to be specifically "antigenic" would tend to *enhance* the induction of immunity. Evidence against an immunological interpretation of the Révész effect included demonstration that the effect of homologous LI cells can be simulated by LI cells of an allogeneic tumour (Hewitt *et al.*, 1973), and by fibrin or brain extract (Peters and Hewitt, 1974); we have no reason to suspect that any of these additives would abrogate any specific immune activity conceived to be directed against the viable tumour cells. The following experiment, using a system in which marked allogeneic immunity was known to be present, yielded results which contradict the assertion that LI cells act by abrogating immune responses: a suspension of foreign (allografted) tumour cells was assayed with or without an added preponderance of their homologous LI cells; in both assays all the tumours which appeared underwent eventual regression; however, the addition of LI cells *increased* by 100-fold the number of viable cells required to obtain temporary growths and hastened regression ot the tumours that did appear. As expected, the additional antigenic material represented by LI cells did not inhibit, but enhanced, the exertion of host immunity.

Reduction of the $TD_{50}$ for syngeneically transplanted tumour cells is also effected by prior exposure of the recipients to sublethal doses of whole body irradiation (WBI). Table III shows the results of parallel assays of viable tumour cells in normal and irradiated recipients. In the case of all of the 5 tumours examined, the $TD_{50}$ was significantly lower in the irradiated mice. It is true that WBI is a powerful suppressant of allograft immunity; but that is no reason at all to ascribe all the effects of WBI, including that shown in Table III, to immunosuppression. The systemic effects of WBI are severe and protean; the doses of WBI we have used may be expected to kill about 95% of the proliferating cells in the body, and the expression of this damage results in flooding of the system with the products of this massive destruction, from which considerable secondary effects are to be expected. However, since repopulation of the depleted cell populations is known to occur, such secondary effects would be of limited duration. Peters (1975), using our CBA Carcinoma "N.T." system, has shown that CBA recipients having *sustained* immunosuppression induced by thymectomy and exposure to WBI several months previously to their use in an assay of this tumour, yield a $TD_{50}$ not significantly different from that given by intact recipients. Evidence of the persistence and intensity of the immunosuppression was given by Peters' demonstration that similarly treated CBA mice were as fully receptive of allografted tumour cells (of WHT Sq. Carcinoma "G") as isogeneic WHT mice. This immaculate evidence

TABLE III.—*Results of Parallel Assays of Tumour Cells in Normal and WBI Mice*

| Tumour | $TD_{50}$ (cells) Normal mice (A) | WBI mice (B) | Reduction factor (A/B) |
|---|---|---|---|
| CBA Sarcoma "F" (14) | 81 | 6 | 13·5 |
| WHT Osteosarcoma I (17) | 182 | 6·6 | 27·6 |
| WHT Sq. Carcinoma "G" (19) | 1096 | 347 | 3·2 |
| CBA Carcinoma "N.T." (24) | 2900 | 100 | 29 |
| | 7900 | 64 | 123 |
| | 3000 | 15 | 200 |
| WHT Osteosarcoma II (27) | 13,200 | 3090 | 4·3 |

clearly indicates that the reduction of $TD_{50}$ observed in WBI mice is due to effects of the irradiation which are quite distinct from immunosuppression.

We conclude from the evidence given in this section that the high $TD_{50}$ values obtained for many tumours are not to be taken as signifying their immunogenicity. We support this conclusion by extensive evidence that none of the many influences which reduce these high values are to be described as immunosuppressive.

We would observe here that we cannot at all reconcile the range of $TD_{50}$ values shown in Table I with an assertion of Southam (1968), that the *minimum* number of " living " tumour cells required for successful transplantation is between $10^4$ and $10^6$, and that this range applies to autografts, isografts, allografts and xenografts.

*Assay data as evidence of the homogeneity of tumour recipients*

As described previously, our assays of solid tumour cell suspensions have involved the injection of a selected series of dilutions into groups of 4 or more mice, each receiving 4 s.c. injections. With the largest mean cell number per site, 100% of the sites are expected to grow tumours; groups of mice receiving progressively smaller numbers of cells yield a falling percentage of takes. If the mice used for assay included individuals having an atypical systemic resistance to the tumour, we should expect to find occasional uniformly injected groups in which one mouse had 0/4 takes while all its fellows had 4/4 takes; a review of our records of 280 assays of various tumours revealed not a single instance of such a coincidence. What is not uncommonly observed is the coincidence of a 0/4 and a 4/4 mouse within a group in which the other mice had 1 to 3 tumours in their injected sites; a certain probability of this occurrence is to be expected near the end-point of an assay, even when the mice are assumed to be homogeneous in their receptivity; indeed, the frequency of such 0/4 : 4/4 coincidence would be greatest in groups having an overall incidence of 50% takes. Following this consideration, we analysed the records of the 280 assays, comprising data for 8 different tumours and 1400 uniformly injected groups, and found 23 groups in which the 0/4 : 4/4 coincidence had occurred; the overall incidence of tumours was determined in these groups, and the values were found to lie in the narrow range 30% to 67%; the mean overall incidence was 50%. Thus, analysis of this very large volume of data revealed no evidence that 0/4 mice were occurring with a frequency that suggested they represented individuals with aberrant systemic " resistance ". There was evidently no suggestion of genetic divergence among the experimental mice in respect of their compatibility with these tumours.

### FAILURE OF ATTEMPTS TO IMMUNIZE MICE AGAINST ISOGENEICALLY TRANSPLANTED TUMOURS OF SPONTANEOUS ORIGIN

A need to identify and quantitate any possible contribution of host immune influences to the results of our radiobiological experiments encouraged us to submit a proportion of our tumour systems to formal tests for tumour immunogenicity. We emphasize that the indication for such examination was most often the emergence of unexpected findings which might be given an immunological interpretation. For example, WHT Ascites Leukaemia I (3) yielded a much higher $TD_{50}$ by s.c. assay than by i.p. assay.

Many " immunizing " techniques have been recommended and as many have been condemned as inadequate. We have used multiple injections of LI homologous tumour cells; induction of immunity has been sought by the performance of parallel assays of viable tumour cells in treated and normal mice; the pattern of these assays permitted comparison of tumour incidence at 4 or 5 levels of challenge. Potentiating adjuvants have not been used because they could have complicated our findings; we have referred in previous sections to numerous minor influences which can *reduce* the $TD_{50}$; a similar nonspecific effect exerted by an adjuvant could conceal the small *rise* in $TD_{50}$ which we sought to reveal. " Immunization " by growth and ablation of tumour entails a considerable risk of loss of treated animals (by metastasis) before the observation period required for the assay has elapsed. Indeed, we suggest that this hazard is more likely to be avoided, and the experiment completed, in the case of tumours that *are* immunogenic and have their metastases suppressed—which may explain the superiority claimed for this technique. However, growth and surgical ablation of some of our tumours have been undertaken in the course of our studies of metastasis: our observation of a frequency of local recurrence or metastasis after such treatment is evidence of its failure to induce resistance in those systems.

Suspensions of tumour cells to be used for putative immunization have been prepared by our usual technique; the cells are killed by exposure to 8000–9000 rad $^{60}Co$ $\gamma$-rays. In a great many experiments with a variety of tumours we have injected large doses of LI tumour cells into isogeneic mice and have observed them for one year: none have grown tumours. Since the dose of radiation used does not significantly inactivate viruses, the long period of observation of these mice permits us to exclude subcellular transmissibility of the tumours used.

Table IV shows for 7 of our tumours the results of parallel assays of tumour cells in normal mice and in mice which had been injected s.c. or i.p. with 2 approximately equal doses of LI cells given at specified intervals before challenge with viable cells. In not a single case was the $TD_{50}$ higher in treated than in control mice; indeed, " immunization " usually *decreased* the TD50 by a factor of about 3·0.

The doses of LI cells given depended on the yield of cells that could be obtained but in terms of total dose of LI cells per unit body weight, the doses we gave exceeded the doses commonly given clinically in " immunotherapy " trials. For example, in the case of WHT Ascites Leukaemia I (3) the total dose of LI cells was equivalent to clinical administration of 40 g of sterilized solid tumour; the minimum dose we used was equivalent to about 2·0 g ($>10^9$ cells).

We reject a suggestion that the reduction of $TD_{50}$ we usually observed in " immunized " mice signifies " immunological enhancement ": the antigens concerned in enhancing phenomena are primarily alloantigens and not tumour specific antigens (Snell, 1970).

### MISCELLANEOUS OBSERVATIONS CONCERNING THE NON-IMMUNOGENICITY OF TUMOURS OF SPONTANEOUS ORIGIN

The experiments to be reported in this section were not necessarily undertaken to examine a suspicion of immunogenicity in the systems used. Nevertheless, they

TABLE IV.—*Results of Parallel Assays of Tumour Cells in Normal Mice and Mice Putatively " Immunized " with Homologous LI Cells*

| Tumour | Serial passage | Total LI cells ($\times 10^{-6}$) | Intervals* (days) | $TD_{50}$ (cells) Normal mice (A) | $TD_{50}$ (cells) " Immunized " mice (B) | Reduction factor (A/B) |
|---|---|---|---|---|---|---|
| CBA Sq. Carcinoma " D " (9) | 82 | 1·3 | 18/11 | 10 | 10 | 1 |
| | 289 | 1·5 | 15/8 | 25 | 10 | 2·5 |
| WHT Fibrosarcoma (10) | 123 | 3·1 | 14/7 | 17 | 5 | 3·4 |
| CBA Sq. Carcinoma I (13) | 61 | 1·3 | 15/8 | 30 | 5 | 6 |
| WHT Asc. Leukaemia I (3) | 140 | 13 | 16/10 | 195 | 89 | 2·2 |
| CBA Fibrosarcoma (18) | 208 | 3·2 | 19/13 | 560 | 190 | 3 |
| CBA Carcinoma " N.T. " (24) | 30 | 0·6 | 17/10 | 7900 | 2400 | 3·3 |
| WHT Carcinoma " N.-C. " (20) | 19 | 0·7 | 19/12 | 1300 | 480 | 2·7 |

* Times before challenge, of 1st and 2nd doses of LI cells.

TABLE V.—*Comparative $TD_{50}$ Values for Assay of Tumours in $F_1$ Mice and Syngeneic Mice*

| Tumour | $F_1$ Cross | $TD_{50}$ (cells) Hybrid mice | $TD_{50}$ (cells) Syngeneic mice* |
|---|---|---|---|
| CBA Leukaemia " Th " (2) | CBA × Albino | 7·6 | (0·71–2·8) |
| WHT Carcinoma " M.T. " (8) | WHT × CBA | >1000 | (4·8–23) |

* Data from Table I.

contribute valuable evidence in support of our contention that our tumours are not immunogenic. It is important to state that we have omitted no observations which go against our contention.

*Assay of tumours in* $F_1$ *hybrids*

From our failure to demonstrate elevation of the $TD_{50}$ in " immunized " mice (Table IV) we asserted that the tumours used were not immunogenic. The assertion requires demonstration that the $TD_{50}$ does reflect minor histocompatibility differences in a transplant system. Such a minor difference prevails when a tumour of origin in a homozygous strain is transplanted to an $F_1$ hybrid between the strain of origin and any foreign strain. The incompatibility is minor because failures to take, or regressions, do not generally occur; indeed, many investigators regularly use $F_1$ hybrids in their tumour studies for logistic convenience. Table V shows the results of assays of 2 of our tumours in $F_1$ hybrids having one parent of the strain of origin. Although syngeneic preference was more evident in the case of the carcinoma, in both cases the $TD_{50}$ is higher in the $F_1$ hybrids than in isogeneic mice. Snell (1958) successfully used immunization with normal tissue cells followed by tumour cell assays to detect weak histocompatibility differences imposed by small specified genetic differences between mouse strains. We conclude that our failure to demonstrate immunogenicity by our " immunization " attempts cannot be ascribed to insensitivity of the challenge assay.

We would add here that $F_1$ hybrid mice are not uncommonly used as recipients in tumour studies given tumour immunological significance. It is clear that such systems may entail artefactual immunity in the form of *allogeneic inhibition* (*i.e.* the converse of *syngeneic preference*).

*Failure to demonstrate an effect on isogeneic tumour transplants of simultaneous exertion by the hosts of induced nonspecific immunity*

Many experimental studies and clinical trials have been undertaken which proceed from a hypothesis that nonspecific stimulation of the immunological resources of the host may restrain the growth or induce the regression of autochthonous tumours that have previously progressed in the host (see Currie, 1974). It seemed to us that an experiment designed to maximize the opportunity for demonstration of such an effect would ensure that: (i) the tumour cells would be exposed to any immune influence while they were few in number and fully accessible—soon after their injection; (ii) any influence on the cells would be evaluable quantitatively; and (iii) the type of immunity to be nonspecifically stimulated would be cell mediated. Accordingly, the following experiment was carried out.

Half of a group of 40 CBA mice were immunised by s.c. injection into the nape of 2 doses of *viable* cells of the foreign tumour, WHT Carcinoma " M.T. " (8); the total inoculum, of $1{\cdot}75 \times 10^6$ cells, gave rise to moderate-sized allograft tumours which later began to regress. The immunized and untreated mice were used 11 days after the second immunising injection as recipients in parallel assays of a suspension of viable cells of CBA Carcinoma " N.T. " (24). A mouse in any group in the assays received 4 ventral s.c.

inocula containing the same specified number of CBA tumour cells; however, in all groups of both assays, the 2 inocula on the left side of a mouse contained CBA tumour cells only, whereas the 2 inocula on the right side contained the same number of CBA tumour cells mixed with $10^5$ LI cells of the foreign (WHT) carcinoma cells. Thus, the immunized and unimmunized mice provided data for 4 simultaneous assays, giving $TD_{50}$ values as shown in Table VI. It is seen that, in the unimmunized mice, CBA tumour cells alone gave a typical $TD_{50}$ value for this tumour; the addition of WHT tumour cells to the inocula significantly reduced the $TD_{50}$ (by a factor of 14). In the immunized mice, the $TD_{50}$ for CBA tumour cells alone was not significantly different from that in the unimmunized mice; the addition of WHT cells to the inocula again reduced the $TD_{50}$, but by a smaller factor, of 4.

It is evident that involvement of the recipients in reactivity against the foreign tumour did not increase the $TD_{50}$, even when the antigenic material was intimately mixed with the isogeneic tumour cells.

Molomut *et al.* (1955), in a rather similar experiment, also failed to show any effect on tumour take or growth of exertion of an *allergic* inflammatory response at the site of the inoculum. In their experiments, the antigen used to immunize, and added to the inocula of tumour cells, was ovalbumin. They used 2 tumours which were both chemically induced and also allografted, so that in their case the nonspecific allergic response had failed even to stimulate an existing level of immunogenicity.

TABLE VI.—*$TD_{50}$ Values for Cells of CBA Carcinoma " N.T. " Assayed with or without Admixed LI WHT Tumour Cells in CBA Mice Immunized or Not Immunized against the WHT Tumour*

| | WHT cells in inocula | |
|---|---|---|
| Recipients | No | Yes |
| Not immunized | 7200 | 510 |
| Immunized | 6500 | 1700 |

Thus, our experiment provided no support whatever for the hypothesis underlying a technique of " immunotherapy " that has been, and continues to be, widely employed in clinical trials. It is commonly insisted that such clinical therapy is expected to influence only small residual populations of tumour cells: this requirement was simulated by the limited and graded inocula of tumour cells used in our assays.

*Effect of admixed bacteria on tumour growth from a limited inoculum of tumour cells*

Live or killed pathogenic bacteria give rise to an acute inflammatory response confined initially to a site of subcutaneous injection. The following experiments examine the effect on tumour take frequency from limited inocula of isogeneic tumour cells of their early involvement in such acute inflammatory response. In 3 separate experiments, approximately 300 viable cells of CBA Carcinoma " N.T. " (24) were injected s.c. in multiple sites into 2 groups of mice; in one group, the tumour cells were injected alone and in the other they were mixed with an equal volume of a dense suspension of bacteria harvested from confluent growths on blood-agar plates. The 3 experiments were distinguished by the different bacterial suspensions used. In the case both of partly viable and of heat-killed coagulase-positive *Staphylococcus aureus*, temporary abscesses were produced at the injection sites between 5 and 13 days after injection. From the results of these experiments (Table VII) it is seen that in all cases the additive greatly increased the frequency of tumours. It is evident that tumour growth was not inhibited by the intensive nonspecific systemic immunostimulation or by local involvement of the tumour cells in the pyogenic response or its sequelae. It is appreciated, however, that this type of immune response is not at all characteristic of that mounted against foreign tissue cells.

TABLE VII.—*The Effect of Bacterial Additives on the Tumour-take Frequency of Small Inocula of Cells of CBA Carcinoma "N.T."*

| No. of tumour cells | Bacterial additive | Tumours/sites injected: No additive | Tumours/sites injected: With additive |
|---|---|---|---|
| 250 | *Staph. aureus.* (part viable)* | 0/48 | 34/44 |
| 300 | *Staph. aureus.* (heat-killed) | 0/46 | 30/44 |
| 360 | *E. coli* (heat-killed) | 0/48 | 47/48 |

* This preparation was exposed to heat treatment intended to sterilize it (60°C × 1 h), but some organisms were found to have survived.

*Failure to inhibit the isogeneic transplantation of leukaemia cells by their admixture with a massive preponderance of spleen cells from specifically " hyperimmunized " mice*

The experiment to be described here was undertaken during attempts to explain anomalous data concerning the radiosensitivity of leukaemia cells irradiated *in vivo* in the infiltrated spleens of leukaemic mice. The results obtained are very significant within the context of this section.

A number of CBA mice each received i.p. a total dose of $2 \cdot 4 \times 10^7$ LI cells of the syngeneic Leukaemia " S1 " I (4) given in 4 doses at about weekly intervals. One week after the final dose, the spleens of the treated mice (which were not enlarged) were pooled and macerated to yield a dense suspension of (mainly) lymphocytes. Viable leukaemia " S1 " cells, with or without admixture with the " hyperimmune " spleen cells were then assayed in parallel in normal CBA mice. The $TD_{50}$ values obtained were 1·1 cells without added spleen cells, and 3·5 cells with spleen cells. In the latter assay, at the end-point, each leukaemia cell was in intimate mixture with a preponderance of $0 \cdot 5 \times 10^6$ spleen cells. Not only are the $TD_{50}$ values not significantly different, but mice receiving equal numbers of leukaemia cells in the 2 assays became sick after almost identical latent periods. It is clear that the spleen cells from " hyperimmunized " mice had exerted no cell killing or growth restraining effect on the very small number of leukaemia cells with which they were mixed. It should be added that each mouse in the assay of the mixed cell population had received $2 \times 10^6$ splenic lymphocytes; and the conditions of the experiment were such that any immune properties that had been generated in them would have been adopted by the recipients in the assay (Mitchison, 1953). Thus, this extremely sensitive technique failed to give any indication whatever that intensive " hyperimmunization " had conferred any specific or nonspecific immune properties on the cells donated, whether these were to act by direct contact with the target cells or after their systemic distribution. It is difficult to believe, in the light of this experiment, that the scanty infiltration of lymphocytes found histologically round some clinical tumours are exerting the tumour restraint that has been ascribed to them by innumerable authors during the last sixty or more years (see Sutherland, 1960).

*Comparative growth of a spontaneous tumour as an autograft in the mouse of origin and as an isograft*

Owing to the relatively infrequent occurrence of spontaneous tumours in low-cancer strain mice, the opportunity to compare auto- and isografts at the first passage rarely arises or is taken. Observations made at this stage of a malignancy are of special importance because it is commonly suggested that serial transplantation may be associated with loss of certain immunological or other characteristics which are peculiar to the original host–tumour relationship.

An ex-breeder CBA female aged 14 months was seen to have an ulcerated 6 × 2 mm tumour plaque in the abdominal skin. This was observed over a period of about 70 days, during which it grew slowly and progressively; at this time the adjacent inguinal node was found to be enlarged. The node and the primary tumour were radically excised under ether anaesthesia. The node, which was about 5 mm diameter, was cut into equal quadrants; one was autotransplanted into a subcutaneous pocket prepared in the flank of the donor opposite to that of the site of tumour excision; another was similarly transplanted to a female CBA taken at random from the stock colony; the remaining quadrants were used for histological study. At the time of grafting it will be appreciated that the donor–recipient was distinguished from the normal recipient in having had several months' contact with the malignancy arising in it; it was therefore long exposed to any immunizing potentiality of the tumour. Observation of the grafts in the 2 hosts showed that both gave rise to just palpable nodules on the same day and subsequently grew at indistinguishable rates.

On the same day as the above grafts were made, a cell suspension was prepared from the primary tumour and assayed in CBA mice: the $TD_{50}$ was exceptionally high ( $>$ 11,000 cells), but this high value was corroborated by a later assay done at the 13th passage. The tumour appears in Table I as CBA Sq. Carcinoma II (26).

Our conclusion from these unusual observations is that prolonged growth of the carcinoma in the mouse of origin was not associated with development of any change in the host that was manifested by a resistance to regrafting relative to the receptivity of an isogeneic unconditioned animal.

*Immunological significance of experiments in lymphnodal metastasis*

In the course of our above studies we have had occasion to transplant isogeneically whole regional lymph nodes draining transplanted tumours of spontaneous origin. In a previous publication (Hewitt and Blake, 1975) we reported that, in the case of WHT Sq. Carcinoma " D " (9), about 40% of such transplanted nodes gave rise to tumours. We submitted evidence to show that the node transplants were disclosing the presence in the node of a small number of tumour cells which were passing through it, very few of which would be destined to give rise to a progressive nodal metastasis if left *in situ* in tumour-excised mice. We have now confirmed in a variety of further systems that regional node transplants commonly yield tumours. These data are to be reported in detail elsewhere but we can summarize these results here by referring to tumours by their serial numbers as given in Table I and giving the percentages of transplanted nodes which yielded tumours: (9) 37; (18) 25; (19) 27; (24) 17; (20) 95; (26) 60. Of incidental interest is that the 2 highest percentages of positive node transplants were given by tumours having large $TD_{50}$ values.

In experiments with WHT Sq. Carcinoma " D " (9) we found also that the frequency of tumours from transplanted nodes was not significantly less for autotransplants than for isotransplants.

The above findings have a considerable implication in the context of tumour immunology. They show that a small number of tumour cells are not restrained in their potential for growth by prolonged contact with an overwhelming preponderance of lymphocytes which have been " sensitized " in the donor mouse by the growth of tumour in the region they drain. This contact is retained in the transplantee; moreover, because the node is transplanted isogeneically, any immune faculties in these node cells will be adopted by the new host (Mitchison, 1953).

The fact that nodes vary in their ability to give rise to tumours is reasonably explained by whether or not there happens to be a sufficient number of tumour cells in a node at the time of its transplantation. The alternative explanation, that some mice are exerting an immune influence and others not, proclaims genetic heterogeneity among the hosts; but that would be clear evidence of artefactual immunity: specific tumour immunity as conceived implies that the tumour is uniformly immunogenic in all the mice that are nominally syngeneic with the mouse of origin.

DISCUSSION

Most tumour immunology experiments using animal tumour systems are explicitly or tendentiously aimed at providing a foundation for clinical immunotherapy; the intention is either to justify its long historical practice, which has been recently reviewed by Currie (1972) or to refine the methods currently used. The endeavour is sustained by one or other of 2 hypotheses: that such therapy can *induce* immunogenicity in a clinical tumour or that it can potentiate an existing level of immunogenicity which is insufficient to assist control of the disease. The results of our studies, using exclusively tumours of spontaneous origin, provide no support for either hypothesis. In particular, we refer to our demonstration that putative " immunization " with homologous LI tumour cells always increased, never reduced, the capacity of isogeneic hosts to support tumour growth from very small inocula of viable tumour cells (Table IV). The technique of immunization we employed, whilst it met the requirements of our experiments and, incidentally, simulated the commonest form of clinical " immunotherapy ", may well be deemed to be suboptimal. However, our systems have always been made freely available to tumour immunologists for more intensive study by the application of other methods of enhancing or measuring an immune response. In only one of the several instances in which advantage was taken was any evidence of an immune response obtained. The exception was in respect of one of our leukaemia lines, for which evidence of immunogenicity has been reported (Smith and Scott, 1972); however, this finding can be excluded from our present context because it was disclosed that the immunogenicity was observed only in graftees which were of a substrain different from that in which the leukaemia arose; the demonstration of immunity clearly depended upon the introduction of an artefactual condition.

We are aware that our uniformly negative evidence has to be set beside a very large volume of reported data from animal tumour studies in which an immune response against tumour has been readily demonstrated. We assert that the peculiarity of our extensive experience is attributable to the category of tumour origin which we have exclusively used; and that the positive evidence with which our own conflicts has been obtained almost entirely from animal systems entailing artefactual immunity, as described in our introductory paragraphs.

Tumour systems entailing artefactual immunity do provide a valuable and proper facility for studying phenomena acknowledged to be immunological. However, we suggest that a much more discriminating choice of animal tumour system is obligatory if it is to serve as a model of the common forms of clinical cancer, information from which is intended to improve our understanding of the human disease or to provide a prescription for innovations into clinical therapy. Encouraged by the prevailing demand for closer collaboration between workers in the research laboratory and those in the clinic, authors of animal data increasingly indulge the inclination to draw direct clinical implications from their findings. The indulgence imposes a heavier responsibility than is often realized, for those who may act clinically upon their advice are commonly, and quite excusably, deficient in the knowledge required to assess the validity of an animal model and the propriety of the analogies drawn, and this is a defect of communication which cannot be excluded even where a clinician acts upon his own findings from animal experiments. In Table VIII we cite a small sample of the papers which have reported the results of animal tumour experiments to which clinical significance was given by the authors by direct clinical application, by discussion in a clinical context or by publication in a journal of predominantly clinical readership. Several of these papers have been very widely

TABLE VIII.—*Status of Transplanted Animal Tumour Systems Used in Experiments Discussed in Relation to Immunology of Clinical Cancer*

| Tumour | Origin* (year) | Reference | Clinical context |
|---|---|---|---|
| Leukaemia L 1210 | C-I (1948) | Mathé (1972) | Immunotherapy† |
| Leukaemia E ♀ K1 | V-I (?) | | |
| Leukaemia, Rauscher | V-I (?) | | |
| Sarcoma 180 | A (1914) | Crile (1965) | Analogy for surgical policy (re node retention) |
| Sarcoma 1 | C-I (1947) | | |
| Walker 256 | A (1928) | Gardner and Rosen (1967) | Discussion (re node retention) |
| ? | C-I (?) | | |
| Lymphosarcoma 6C3HED (Gardner) | C-I (1941) | Perez *et al.* (1973) | Discussion (re node retention) |
| BLMC Fibrosarcoma | C-I (~1969) | Hammond and Rolley (1970) | Discussion (re node retention) |
| Fibrosarcoma MC-42 | C-I (?) | Simmons and Rios (1973) | Discussion (use of BCG) |
| Fibrosarcoma MC-43 | C-I (?) | | |
| Fibrosarcoma | C-I (1964) | Haddow and Alexander (1964) | *Lancet* (Radiotherapy + imm.) |
| Lymphosarcoma 6C3HED (Gardner) | C-I (1941) | Powers and Palmer (1967) | Discussion (Radiotherapy + imm.) |
| Fibrosarcoma | C-I (?) | Suit *et al.* (1975) | Discussion (immunotherapy) |
| Leukaemia LSTRA | V-I (?) | Pearson *et al.* (1972) | Discussion (immunotherapy) |
| Leukaemia E ♂ G2 | V-I (?) | Amiel and Berardet (1970) | Discussion (immunotherapy) |
| Fibrosarcoma MC1 | C-I (?) | Currie and Bagshawe (1970) | *Br. med. J.* (immunotherapy) |
| Fibrosarcoma MC6 | C-I (?) | | |

* C-I = chemically-induced; V-I = virus-induced; A = allograft.
† " These experimental results form the basis of the possible clinical application of active immunotherapy. " (Mathé, 1972.)

quoted in support of clinical intentions; yet in no case were the data obtained from an isogeneically transplanted tumour of spontaneous origin; we are not aware that these authors have been able to confirm their findings using more eligible experimental systems.

Many of the terms used to describe an animal tumour system, though correct, can convey unwarranted confidence in its eligibility as a model—at least to the uninitiated: " primary ", " autochthonous " and " isogeneically transplanted " are terms which exclude only frank allografted tumours; they do not exclude systems entailing artefactual immunity attributable to their mode of induction or to genetic diversity associated with a substrain difference between the animal in which a tumour arose and the graftees used. The term " spontaneous " is commonly, and quite incorrectly, used to describe the characteristic malignancies arising in high leukaemia or mammary tumour mouse strains such as AKR or C3H respectively; these malignancies are, of course, *induced* by vertically transmitted exogeneous viruses, albeit in immunologically tolerant hosts. We suggest that " spontaneous " be reserved to describe tumours arising in circumstances where no oncogenic agency has been proved to be involved; the category deserves distinction for the immunological peculiarity we have described. To disallow all usage of " spontaneous " on metaphysical grounds that all events have causes would be merely to invite the neologism we should then require.

Enchantment of experimenters with the general theory of tumour immunity has, in our view, led to the too facile attachment of immunological interpretations to complex manifestations of cancer biology deserving of much broader consideration. The effect has been to treat such interpretations as final rather than provisional and so to abate the scepticism that inspires further investigation. We have already referred to the persistence of the theory that the facilitation of tumour graft acceptance effected by prior WBI of recipients is solely due to immunosuppression; yet it has been shown by van den Brenk, Sharpington and Orton (1973) that, even in allogeneic systems, several non-immunological factors contribute to this effect of WBI. The appealing immunological connotations attaching historically to BCG have, likewise, encouraged an exclusively immunological interpretation of its effects on tumour growth. The severity of its systemic effects, the peculiar technical conditions required for demonstration of its effect on tumour growth, and the fact that such effects can be inhibiting or enhancing (Baldwin and Pimm, 1973) invite more liberal interpretation. Caution is equally required in interpreting the apparently favourable effect of BCG in clinical "immunotherapy" (Crowther *et al.*, 1973). Demonstration of clinical advantage does not necessarily ratify the theory instigating a trial.

We suspect that the almost exclusive resort of tumour immunologists to chemically induced, virus-induced or allografted tumours is calculated to eke out evidence supporting the general theory of effective tumour immunogenicity which otherwise might fail to attract the very large attention it receives. We venture to suggest that expansion of an already vast volume of literature would be considerably restrained if publication of papers drawing clinical implications from animal experiments in this field were made conditional upon the use of tumour systems free from the more obvious stigmata of artefactual immunity.

Almost half a century ago the pertinence of the contemporary data from animal tumour studies was questioned in the more robust and critical style which was then permissible (Woglom, 1929; Mottram, 1930), but investigators were at that time severely restricted in respect of the animal tumours and related technical facilities available to them. Whereas the deprivations of the past can only be regretted, the motivations influencing free choice at the present time deserve to be questioned.

We are grateful to Dr H. A. S. van den Brenk for the benefit of his extensive clinical and laboratory experience in many discussions, and for his review of the script, to Miss Carol Dear and Mrs Patricia Whitman for assistance in the care of animals, and to Mrs Karen Jepson for preparation of the manuscript. Dr D. C. Roberts and the Registry and Information Service for Experimental Tumours (Imperial Cancer Research Fund) provided indispensable assistance in tracing the origin of the tumours we have directly or indirectly referred to.

The work was supported exclusively by the Cancer Research Campaign.

### REFERENCES

AMIEL, J. L. & BERARDET, M. (1970) An Experimental Model of Active Immunotherapy Preceded by Cytoreductive Chemotherapy. *Eur. J. Cancer*, **6**, 557.

BALDWIN, R. W. & PIMM, M. V. (1973) BCG Immunotherapy of Rat Tumours of Defined Immunogenicity. *Natn. Cancer Inst. Monog.*, **39**, 11.

BERENBAUM, M. C. (1964) Effect of Carcinogens on Immune Processes. *Br. med. Bull.*, **20**, 159.

COLLINS, V. P., LOEFFLER, R. K. & TIVEY, H. (1956) Observations on Growth Rates of Human Tumors. *Am. J. Roentg.*, **76**, 988.

CRILE, G. JR (1965) Rationale of Simple Mastectomy Without Radiation for Clinical Stage I Cancer of the Breast. *Surgery Gynec. Obstet.*, **120**, 975.

CROWTHER, D., POWLES, R. L., BATEMAN, C. J. T., BEARD, M. E. J., GAUCI, C. L., WRIGLEY, P. F. M., MALPAS, J. S., FAIRLEY, G. H. & BODLEY SCOTT, R. (1973) Management of Adult Acute Myelogenous Leukaemia. *Br. med. J.*, i, 131.

CURRIE, G. A. (1972) 80 Years of Immunotherapy: A Review of Immunological Methods Used for the Treatment of Human Cancer. *Br. J. Cancer*, **26**, 141.

CURRIE, G. A. (1974) *Cancer and the Immune Response*. London: Edward Arnold.

CURRIE, G. A. & BAGSHAWE, K. D. (1970) Active Immunotherapy with *Corynebacterium parvum* and Chemotherapy in Murine Fibrosarcomas. *Br. med. J.*, i, 541.

DUNHAM, W. B. & WAYMOUTH, C. (1972–73) *44th Annual Report of the Jackson Laboratory*, p. 20.

FOLEY, E. J. (1953) Antigenic Properties of Methylcholanthrene-induced Tumors in Mice of the Strain of Origin. *Cancer Res.*, **13**, 835.

GARDNER, G. & ROSEN, R. (1967) The Effect of Lymphadenectomy on Tumor Immunity in the Rat. *Surgery Gynec. Obstet.*, **125**, 351.

GROSS, L. (1943) Intradermal Immunization of C3H Mice Against a Sarcoma that Originated in an Animal of the Same Line. *Cancer Res.*, **3**, 326.

HADDOW, A. & ALEXANDER, P. (1964) An Immunological Method of Increasing the Sensitivity of Primary Rat Sarcomas to Local Irradiation with X-rays. *Lancet*, i, 452.

HAMMOND, W. G. & ROLLEY, R. T. (1970) Retained Regional Lymph Nodes: Effect on Metastases and

Recurrence after Tumour Removal. *Cancer, N.Y.*, **25**, 368.
HEWITT, H. B. (1958) Studies of Dissemination and Quantitative Transplantation of a Lymphocytic Leukaemia of CBA Mice. *Br. J. Cancer*, **12**, 378.
HEWITT, H. B. (1961) Transplantation of Murine Leukaemia by Unconventional Routes. *Nature, Lond.*, **191**, 1213.
HEWITT, H. B. (1966) The Effect on Cell Survival of Inhalation of Oxygen under High Pressure during Irradiation *in vivo* of a Solid Mouse Sarcoma. *Br. J. Radiol.*, **39**, 19.
HEWITT, H. B. & BLAKE, E. R. (1975) Quantitative Studies of Translymphnodal Passage of Tumour Cells Naturally Disseminated from a Non-immunogenic Murine Squamous Carcinoma. *Br. J. Cancer*, **31**, 25.
HEWITT, H. B., BLAKE, E. & PORTER, E. H. (1973) The Effect of Lethally Irradiated Cells on the Transplantability of Murine Tumours. *Br. J. Cancer*, **28**, 123.
KLEIN, G. (1970) Immunological Factors Affecting Tumour Growth. *Br. med. J.*, iv, 418.
MARCHANT, J. (1968) Antigenic Properties of Spontaneously-occurring Tumours of Mice. *46th Ann. Rep. Br. Empire Cancer Camp.*, p. 250.
MATHÉ, G. (1972) Immunological Approaches of Leukaemia Treatment. *Annls Inst. Pasteur, Paris*, **122**, 855.
MITCHISON, N. A. (1953) Passive Transfer of Transplantation Immunity. *Nature, Lond.*, **171**, 267.
MOLOMUT, N., SPAIN, D. M., KREISLER, L. & WARSHAW, L. J. (1955) The Effect of an Allergic Inflammatory Response in the Tumor Bed on the Fate of Transplanted Tumors in Mice. *Cancer Res.*, **15**, 181.
MOTTRAM, J. C. (1930) Utilisation of Immunity in Treatment of Cancer. *Lancet*, i, 961.
PEARSON, J. W., PEARSON, G. R., GIBSON, W. T., CHERMANN, J. C. & CHIRIGOS, M. A. (1972) Combined Chemicoimmunostimulation Therapy against Murine Leukemia. *Cancer Res.*, **32**, 904.
PEREZ, C. A., STEWART, C. C., PALMER-HANES, L. A. & POWERS, W. E. (1973) The Role of the Regional Lymph Nodes in the Cure of a Murine Lymphosarcoma. *Cancer, N.Y.*, **32**, 562.
PETERS, L. J. (1975) Enhancement of Syngeneic Murine Tumour Transplantation by Whole Body Irradiation—a Non-immunological Phenomenon. *Br. J. Cancer*, **31**, 293.
PETERS, L. J. & HEWITT, H. B. (1974) The Influence of Fibrin Formation on the Transplantability of Murine Tumour Cells: Implications for the Mechanism of the Révész Effect. *Br. J. Cancer*, **29**, 279.
PORTER, E. H., HEWITT, H. B. & BLAKE, E. R. (1973) The Transplantation Kinetics of Tumour Cells. *Br. J. Cancer*, **27**, 55.
POWERS, W. E. & PALMER, L. A. (1967) Cellular Sensitivity and Tumor Curability. *Natn. Cancer Inst. Monog.*, **24**, 169.
PREHN, R. T. (1963) Function of Depressed Immunologic Reactivity during Carcinogenesis. *J. natn. Cancer Inst.*, **31**, 791.
PREHN, R. T. & MAIN, J. M. (1957) Immunity to Methylcholanthrene-induced Sarcomas. *J. natn. Cancer Inst.*, **18**, 769.
RÉVÉSZ, L. (1956) Effect of Tumor Cells Killed by X-rays upon the Development of Admixed Viable Cells. *J. natn. Cancer Inst.*, **20**, 1157.
Report of the Medical Committee of the Society for Investigating the Nature and Cure of Cancer (1806). *Edinburgh med. surg. J.*, **2**, 382.
ROUS, P. (1965) Viruses and Tumour Causation: An Appraisal of Present Knowledge. *Nature, Lond.*, **207**, 457.
SIMMONS, R. L. & RIOS, A. (1973) Comparative and Combined Effect of BCG and Neuraminidase in Experimental Immunotherapy. *Natn. Cancer Inst. Monog.*, **39**, 57.
SMITH, S. E. & SCOTT, M. T. (1972) Biological Effects of *Corynebacterium parvum*: III. Amplification of Resistance and Impairment of Active Immunity to Murine Tumours. *Br. J. Cancer*, **26**, 361.
SMITHERS, D. W. (1962) Cancer: An Attack on Cytologism. *Lancet*, i, 493.
SNELL, G. D. (1958) Histocompatibility Genes of the Mouse. I. Demonstration of Weak Histocompatibility Differences by Immunization and Controlled Tumor Dosage. *J. natn. Cancer Inst.*, **20**, 787.
SNELL, G. D. (1970) Immunologic Enhancement. *Surgery Gynec. Obstet.*, **130**, 1109.
SOUTHAM, C. M. (1968) Factors Influencing the Growth of Tumor Autotransplants. In *The Proliferation and Spread of Neoplastic Cells.* Baltimore: Williams and Wilkins. p. 583.
SOUTHAM, C. M. & BRUNSCHWIG, A. (1961) Quantitative Studies of Autotransplantation of Human Cancer. *Cancer, N.Y.*, **14**, 971.
STJERNSWÄRD, J. (1965) Immunodepressive Effect of 3-methylcholanthrene. Antibody Formation at the Cellular Level and Reaction against Weak Antigenic Homografts. *J. natn. Cancer Inst.*, **35**, 885.
SUIT, H. & KASTELAN, A. (1970) Immunologic Status of Host and Response of a Methylcholanthrene Induced Sarcoma to Local X-irradiation. *Cancer, N.Y.*, **26**, 232.
SUIT, H. D., SEDLACEK, R., WAGNER, M. & ORSI, L. (1975) Radiation Response of C3H Fibrosarcoma Enhanced in Mice Stimulated by *Corynebacterium parvum*. *Nature, Lond.*, **255**, 493.
SUTHERLAND, R. (1960) *Cancer: The Significance of Delay*. London: Butterworth.
SZAKAL, A. K. & HANNA, M. G. (1972) Immune Suppression and Carcinogenesis in Hamsters During Topical Application of 7, 12-Dimethylbenz(a)anthracene. *Natn. Cancer Inst. Monog.*, **35**, 173.
VAN DEN BRENK, H. A. S., SHARPINGTON, C. & ORTON, C. (1973) Macrocolony Assays in the Rat of Allogeneic Y-P388 and W-256 Tumour Cells Injected Intravenously: Dependence of Colony Forming Efficiency on Age of Host and Immunity. *Br. J. Cancer*, **27**, 134.
WOGLOM, W. H. (1929) Immunity to Transplantable Tumours. *Cancer Rev.*, **4**, 129.